# Estimation of the epidemiological burden of HPV-related anogenital cancers, precancerous lesions, and genital warts in women and men in Europe: Potential additional benefit of a nine-valent second generation HPV vaccine compared to first generation HPV vaccines

**DOI:** 10.1016/j.pvr.2015.06.003

**Published:** 2015-06-16

**Authors:** Susanne Hartwig, Jean-Jacques Baldauf, Géraldine Dominiak-Felden, François Simondon, Laia Alemany, Silvia de Sanjosé, Xavier Castellsagué

**Affiliations:** a Department of Epidemiology, Sanofi Pasteur MSD, Lyon, France; b Département de gynécologie et obstétrique, Hôpital de Hautepierre, Strasbourg, France; c IRD UMR216, Mère et enfant face aux infections tropicales, Paris, 75006, France; COMUE Sorbonne Paris Cité, Université Paris Descartes, Faculté des Sciences Pharmaceutiques et Biologiques, Paris, 75270, France; d Cancer Epidemiology Research Program, Institut Català d’Oncologia (ICO)-IDIBELL, CIBERESP, L’Hospitalet de Llobregat, Catalonia, Barcelona, Spain

**Keywords:** AIN, anal intraepithelial neoplasia, CI, confidence interval, CI5, Cancer Incidence in Five Continents, CIN, cervical intraepithelial neoplasia, HPV, human papillomavirus, IARC, International Agency for Research on Cancer, VIN, vulvar intraepithelial neoplasia, VaIN, vaginal intraepithelial neoplasia, HPV, Burden of disease, Cancer, Precancerous lesions, Genital warts, HPV vaccine

## Abstract

**Introduction:**

A second generation HPV vaccine has been developed for the prevention of anogenital cancers and precancerous lesions of the cervix, vulva, vagina, anus and of genital warts due to nine HPV types.

We estimated the annual burden of these diseases attributable to the nine HPV types compared to HPV types from first generation vaccines in women and men in Europe.

**Material and methods:**

Incidence rates from the IARC database, cancer registries, the literature and Eurostat population data were used.

The burden attributable to the HPV types targeted by both vaccines was estimated by applying the relative contribution of the respective HPV types from epidemiological studies.

**Results:**

In 2013, the number of new anogenital HPV-attributable cancers was 44,480 with 39,494 of these cases related to second vs. 33,285 to first generation vaccine types.

Among the 284,373 to 541,621 new HPV-attributable anogenital precancerous lesions 235,364–448,423 and 135,025–256,830 were estimated to be related to second and first generation vaccine types, respectively.

The annual number of new genital warts was 753,608–935,318, with 90% related to HPV6/11.

**Conclusions:**

These data demonstrate how the large public health impact that was achieved by the first generation HPV vaccines could be further increased by second generation vaccines.

## 1 Introduction

The discovery of human papillomavirus (HPV) as the necessary cause of cervical cancer has led to the development of different prophylactic HPV vaccines. The International Agency for Research on Cancer (IARC) has identified twelve HPV types as carcinogenic to humans: HPV16/18/31/33/35/39/45/51/52/56/58/59 [1]. In addition to cervical cancer, HPV is responsible for a significant proportion of cancers and precancerous lesions of the vulva, vagina, and anus in women; cancers and precancerous lesions of the anus and penis in men; and a subset of head and neck cancers and genital warts in both sexes.

Two HPV vaccines have been licensed so far in Europe: the quadrivalent HPV vaccine, Gardasil^®^ 9 (Sanofi Pasteur MSD)/Silgard® (Merck Sharp & Dohme), and the bivalent HPV vaccine, Cervarix® (GlaxoSmithKline Biologicals). Both vaccines have reassuring safety profiles, as demonstrated in clinical trials, and are indicated for the prevention of cervical, vulvar and vaginal premalignant lesions and cervical cancer related to HPV16/18. The quadrivalent HPV vaccine is also indicated against premalignant anal lesions and anal cancer and protects against low-risk HPV6/11, which are responsible for about 90% of genital warts [2] and a fraction of precancerous lesions.

As it takes several decades for HPV infection to progress to cancer, it will be some time before the real effects of current HPV vaccination programs will be seen in terms of a reduced incidence of HPV-related cancers. In the meantime, in countries like Australia, where vaccination coverage is high, a reduction of 77% in the prevalence of infections with the HPV types targeted by the vaccines has been observed in young women [3], along with a 92.6% reduction in the incidence of genital warts (*p*_trend_<0.0001) [4]. Moreover, just 3 years after the implementation of the HPV vaccination program in Australia, the incidence of high-grade cervical precancerous lesions in women under 18 years of age decreased, from 0.85% in 2006 (1 year before vaccine introduction) to 0.22% in 2009 (*p*=0.003) [5]. This ecological observation was confirmed in two recent studies. One was a data-linkage study [6] that reported an adjusted vaccine effectiveness of 47.5% (95% confidence interval [CI]: 22.7–64.4%) to prevent cervical intraepithelial neoplasia (CIN) grade 3/adenocarcinoma in situ or worse among women who received all required doses of the quadrivalent vaccine (i.e., were fully vaccinated). The other study [7] reported a vaccine effectiveness of 46% (95% CI: 33–57%) for histologically confirmed high-grade cervical precancerous lesions among young women who were fully vaccinated before they initiated cervical cancer screening. Additionally, as a reflection of herd immunity, there has been a significant reduction in the frequency of genital warts in young men. In Australia, where there is an estimated 83% coverage of the first dose of HPV vaccine in girls aged 12–13 years, the reduction in the proportion of new genital warts cases among young men was in the order of 60% [8], [9]. In 2013, Australia became the first country to implement a school-based vaccination program for boys aged 12–13 years. Currently the United States, Ireland, New Zealand, and Canada are also recommending HPV vaccination for boys.

After HPV16/18, high-risk HPV31/33/35/45/52/58 are the six most frequently detected HPV types in invasive cervical cancer worldwide [10]. Merck has developed a second generation nine-valent HPV L1 virus-like particle vaccine (Gardasil^®^ 9), which aims to protect against the seven high-risk HPV types (HPV16/18/31/33/45/52/58) most frequently responsible for cervical cancer development worldwide, and the low-risk HPV types (HPV6/11) responsible for about 90% of genital warts. Thus, the nine-valent vaccine is designed to protect against five ‘new’ high-risk HPV types (HPV31/33/45/52/58) that are not targeted by the quadrivalent or the bivalent vaccine. The vaccine is indicated to protect against premalignant lesions and cancers affecting the cervix, vulva, vagina and anus caused by vaccine HPV types as well as genital warts caused by specific HPV types.

In a Phase III study, the nine-valent vaccine prevented 97% of high-grade precancerous lesions of the cervix, vulva, and vagina caused by the five new high-risk HPV types (HPV31/33/45/52/58) [11]. The nine-valent vaccine also generated immune responses to HPV6/11/16/18 that were as good as or better than those generated by the quadrivalent vaccine.

The aim of this study was to estimate the annual burden of selected cancers, precancerous lesions, and genital warts attributable to the HPV types targeted by the second generation nine-valent HPV vaccine Gardasil 9® (high-risk HPV16/18/31/33/45/52/58, low-risk HPV6/11) in women and men in Europe in 2013, and to compare this to the estimated annual burden of the same lesions related to the HPV types targeted by the first generation HPV vaccines.

## 2 Material and methods

### 2.1 Estimation of the annual burden of cancer in Europe

The Cancer Incidence in Five Continents (CI5) database, available on the IARC website [12], contains worldwide data on cancer incidence rates classified by International Classification of Diseases 10th Revision (ICD-10) codes. These data are obtained from regional or national registries, depending on the country, but to be included in CI5 these registries must meet the IARC’s quality criteria, i.e., they must have reliable cancer registry data. The present report includes cancer incidence data from CI5 Volume X, which were collected from 2003 throughout 2007. We selected a total of 32 countries: all 31 countries covered by the European Medicines Agency (Austria, Belgium, Bulgaria, Croatia, Cyprus, the Czech Republic, Denmark, Estonia, Finland, France, Germany, Greece, Hungary, Iceland, Ireland, Italy, Latvia, Liechtenstein, Lithuania, Luxemburg, Malta, the Netherlands, Norway, Poland, Portugal, Romania, Slovenia, Slovakia, Spain, Sweden, and the United Kingdom) plus Switzerland.

**Fig. 1:**
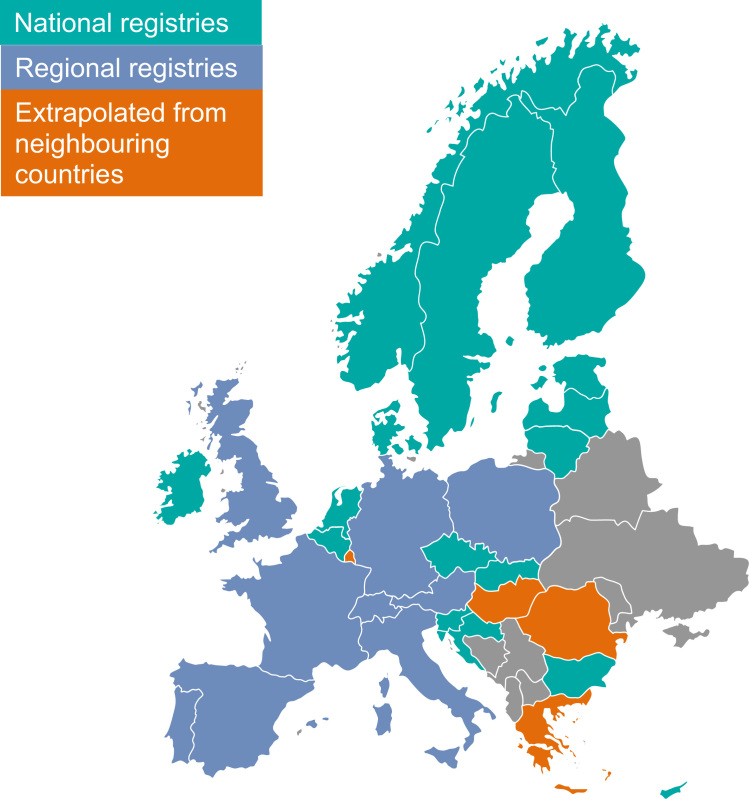
Selected countries: European Medicines Agency region and Switzerland (32 countries).

The information in CI5 Volume X was obtained from national cancer registries for Belgium, Bulgaria, Croatia, Cyprus, the Czech Republic, Denmark, Estonia, Finland, Iceland, Ireland, Latvia, Lithuania, Malta, the Netherlands, Norway, Slovenia, Slovakia, and Sweden. The information in CI5 Volume X was obtained from regional cancer registries for Austria, France, Germany, Italy, Poland, Portugal, Spain, Switzerland, and the United Kingdom (Fig. 1).

To ensure that national populations were adequately represented in countries where only regional cancer registries exist, we assessed the geographical coverage and distribution of these registries.

Five of the 32 countries selected did not have data available in CI5 Volume X, as they did not have reliable cancer registry data: Greece, Hungary, Liechtenstein, Luxemburg, and Romania. We excluded Lichtenstein from our analysis, but for the remaining four countries we extrapolated the age-specific average cancer incidence rates of neighboring countries, or from cancer registries in the same area as the countries selected. The choice of countries used for extrapolation was the same as that in the Globocan database [13]. Thus for Greece data from Bulgaria, Cyprus, and Central Serbia were used; for Hungary data from Austria, Croatia, Central Serbia, Slovakia, and Slovenia were used; for Luxemburg data from French and German cancer registries were used; and for Romania data from Bulgaria, Slovakia, and one regional registry in Romania were used.

In conclusion, our results are referring to a geographical region of 31 European countries.

### 2.2 Estimation of the annual number of new selected HPV-related cancers in Europe

The following HPV-related cancer sites were selected: cervix (ICD-10 code C53), vulva (C51), vagina (C52), and anus (C21, both sexes). We estimated the mean annual number of new cancers at these sites in the selected countries based on the sex- and age-specific cancer incidence data available in CI5 Volume X [14], and extrapolated to the population of each country using 2013 Eurostat population data (for Italy we used 2012 population data, as 2013 data were not yet available) [15] as follows:

$$\mathrm{Total}\;nb\;\mathrm{of}\;\mathrm{new}\;\mathrm{cases}=\frac{\sum_{\mathrm{Countries}}\left\{\sum_{age=0}^{85+}(\mathrm{AIR}\;\mathrm{in}\;\mathrm{male}⁎\mathrm{population}+\sum_{\mathrm{type}\;\mathrm{of}\;\mathrm{cancer}}(\mathrm{AIR}\;\;\text{in}\;\mathrm{female}⁎\mathrm{population}))\right\}}{100,000}$$

where AIR is the age- and sex-specific annual incidence rate, and population is the age- and sex-specific population. The estimated numbers of new HPV-related cancer cases for all the selected countries were then summed to obtain the overall European burden. Sex-specific data were available for anal cancer. However, as preference was given to European data, sex-specific data were not used, as they were not considered robust enough due to the small sample size. When adjusted and crude data were available, preference was given to adjusted data.

The number of new cancers attributable to HPV overall, to HPV16/18, and to HPV16/18/31/33/45/52/58 was then estimated by applying the corresponding cancer site-specific HPV prevalence. Data on HPV prevalence were extracted from the most relevant published cancer site-specific data. To avoid overestimating the prevalence of individual HPV types due to multiple infections, different adjustment methods, which are described in the cited paper for each cancer site, were used.

There are other cancers known to be associated with HPV, including penile cancer in men and a subset of head and neck cancers in both sexes. We did not estimate the burden of these cancers in this work, as the currently licensed HPV vaccines don’t have an indication for cancers at these anatomical sites.

Moreover, the data currently available did not allow us to calculate the burden associated with the seven high-risk HPV types targeted by the nine-valent vaccine. Robust studies to estimate the prevalence of HPV in these cancer sites, and more specifically the attributable fraction of the seven high-risk HPV types targeted by the nine-valent vaccine, are still ongoing.

### 2.3 Estimation of the annual number of new selected HPV-related precancerous lesions in Europe

We performed a literature review to retrieve robust age-specific incidence data for cervical pre-invasive neoplasia CIN2+ (including CIN2, CIN3 and adenocarcinoma in situ (AIS), vulvar intraepithelial neoplasia grades 2 and 3 (VIN2/3), and vaginal intraepithelial neoplasia grades 2 and 3 (VaIN2/3) in Europe.

Nygard et al. estimated the incidence rates of CIN2+ (including CIN2/3 and AIS) per 100,000 woman years in 2004–2006, the period prior to the availability of the quadrivalent vaccine. The estimates were presented by age group and were age-standardized using the European standard population. Data were available for four Nordic countries: Denmark, Iceland, Norway, and Sweden. Moreover, we had access to incidence data on CIN2+ for the period 2009–2011 from the French cancer registry of the Alsace Region, where an organized screening program has been in place since 1994 in Bas–Rhin and since 2001 in Haut–Rhin (unpublished data). Based on the data from these five countries we estimated incidence rate ranges for CIN2+ by age group, which we then extrapolated to the combined female population of the 31 included countries. The study by Nygard et al. [16] also contained incidence data for VIN2/3 and VaIN2/3 by age group from Denmark, Iceland, Norway, and Sweden for the period 2004–2006. However, as only VIN3 and VaIN3 were recorded in Sweden, we excluded the Swedish data and based our estimates on the data from the remaining three countries.

We then estimated the number of CIN2+ (including CIN2/3 and AIS), VIN2/3, and VaIN2/3 cases attributable to the HPV types targeted by the nine-valent vaccine and the quadrivalent vaccine, respectively. For CIN2+ we applied the site-specific overall HPV prevalence, the relative contribution of HPV6/11/16/18, and that of HPV6/11/16/18/31/33/45/52/58, respectively. As no specific European data for CIN2+ have yet been published, the authors of the recently published paper [17] kindly provided us with information specific to Europe. We applied HPV prevalence and type attributable fractions from published literature for VIN2/3 [18] and VaIN2/3 [19], as well as additional data we requested from the authors of these studies. The adjustment methods that were used to account for multiple infections are described in the cited papers for each lesion type.

As no age-specific incidence data are available for anal intraepithelial neoplasia grades 2 and 3 (AIN2/3), we used sex-specific, age-standardized incidence rates from the Danish Registry of Pathology [20] and extrapolated them to the female and the male population, respectively, of all 31 European countries. The estimated annual number of AIN2/3 attributable to HPV overall, HPV6/11/16/18, and HPV6/11/16/18/31/33/45/52/58, respectively, was based on HPV prevalence data extracted from Alemany et al. [19], as well as additional data we requested from the authors of that study.

### 2.4 Estimation of the annual number of new genital warts cases in Europe

To estimate the annual number of new genital warts cases we used the methodology previously described [21]. Briefly, two European publications were identified that, based on their design, provided the most robust incidence data for Europe [22], [23]. Both are retrospective cohort studies carried out using databases, including very large samples of routinely collected data. We extrapolated the value from each publication to the 2013 population of the 31 European countries to provide an estimated range of the annual number of new genital warts cases in women and men in Europe. The estimated prevalence of HPV6/11 of 90% in genital warts was then applied to estimate the number of cases attributable to the HPV types targeted by the nine-valent and quadrivalent vaccines.

## 3 Results

### 3.1 Women

#### 3.1.1 HPV-related cancers in women

##### 3.1.1.1 Cervical cancer

The estimated annual number of new cervical cancers in 2013 was 34,708 (95% bound: 32,640–36,793) in the 31 selected European countries combined. It is generally accepted that HPV infection is necessary for the development of cervical cancer [24], thus 100% of these cases are believed to be HPV-positive. HPV16/18 are the predominant types in cervical cancer, accounting for 72.8% (95% CI: 70.8–74.7) of cases [10]; high-risk HPV16/18/31/33/45/52/58 were estimated to be responsible for 89.0% (95% CI: 87.5–90.3) of cases in Europe (Table 1). Accordingly, a total of 25,267 (95% bound: 23,062–27,528) cases were estimated to be attributable to HPV16/18 versus 30,890 (95% bound: 28,539–33,242) cases attributable to HPV16/18/31/33/45/52/58. The estimated annual number of new cervical cancer cases attributable to the five new HPV types targeted by the nine-valent vaccine (HPV31/33/45/52/58) was thus 5623 (95% bound: 4681–6640) (Table 2).

**Table 1:** Overall HPV prevalence and the relative contribution^a^ of HPV16/18 and HPV16/18/31/33/45/52/58 by cancer site.

Cancer site	ICD-10 code	HPV prevalence %, (95% CI)	HPV16/18 attributable fraction among HPV+ cases %, (95% CI)	HPV 16/18/31/33/45/52/58 attributable fraction among HPV+ cases%, (95% CI)	HPV 31/33/45/52/58 attributable fraction among HPV+ cases%	Reference
Cervix	C53	100	72.8 (70.8–74.7)	89.0 (87.5–90.3)	+ 16.2 (14.6–17.8)	de Sanjosé et al. [10]
Vulva	C51	19.3 (16.7–22.0)	73.6 (66.4–79.9)	84.0 (77.6–89.0)	+ 10.5 (6.2–15.9)	de Sanjosé et al. [18]
Vagina	C52	71.1 (63.2–78.1)	71.2 (61.8–79.6)	85.6 (77.1–91.3)	+ 14.4 (7.9–21.9)	Alemany et al. [25]
Anus	C21	87.6 (81.6–92.1)	87.1 (80.7–92.1)	89.8 (83.8–94.2)	+ 2.7 (0.7–6.8)	Alemany et al. [19]

HPV: human papillomavirus; ICD-10: International Classification of Diseases, 10th Revision; CI: confidence interval.

a Adjusted for multiple infections.

**Table 2:** Estimated mean annual number of new HPV-related cancer cases in women and men in Europe.

Cancer site	*N* of new cancers irrespective of HPV status (95% bound)	*N* of new cancers attributable to HPV (95% bound)	*N* of new cancers attributable to HPV16/18 (95% bound)	*N* of HPV16/18/31/33/45/52/58+ cancers (95% bound)	*N* of cases attributable to additional types (9v vaccine vs 2v/4v vaccine)
Cervical cancer	34,708 (32,640–36,793)	34,708 (32,640–36,793)	25,267 (23,062–27,528)	30,890 (28,539–33,242)	5623 (4681–6640)
Vulvar cancer	9,544 (8,509–10,596)	1,842 (1,377–2,381)	1,356 (903–1,911)	1,547 (1,061–2,124)	193 (71–401)
Vaginal cancer	2,171 (1,676–2,684)	1,544 (1,046–2,107)	1,099 (635–1,685)	1,322 (799–1,927)	222 (69–483)
Anal cancer (F)	4,562 (3,875–5,265)	3,996 (3,151–4,855)	3,481 (2,533–4,477)	3,589 (2,632–4,577)	108 (13–356)
Total (women)	50,985 (47,018–54,983)	42,090 (38,436–45,863)	31,203 (27,319–35,353)	37,347 (33,230–41,609)	6146 (4880–7780)
Anal cancer (M): Total (men)	2,729 (2,195–3,279)	2,390 (1,783–3,025)	2,082 (1,431–2,790)	2,147 (1,487–2,852)	65 (6–226)

Total (both sexes)	53,714 (49,296–58,165)	44,480 (40,294–48,795)	33,285 (28,818–38,055)	39,494 (34,787–44,372)	6210 (4892–7981)
%	100	83	62	74	12

HPV: human papillomavirus; CIN: cervical intraepithelial neoplasia; VIN: vulvar intraepithelial neoplasia; VaIN: vaginal intraepithelial neoplasia; AIN: anal intraepithelial neoplasia; CI: confidence interval.

##### 3.1.1.2 Vulvar cancer

The estimated annual number of new vulvar cancers was 9544 (95% bound: 8509–10,596). Given the overall HPV prevalence in vulvar cancer in Europe of 19.3% (95% CI: 16.7–22.0) [18], 1842 (95% bound: 1377–2381) cases were estimated to be attributable to HPV. The relative contribution of HPV16/18 and HPV16/18/31/33/45/52/58 were estimated at 73.6% (95% CI: 66.4–79.9) and 84.0% (95% CI: 77.6–89.0), respectively [18] (Table 1). After applying these relative contribution estimates, 1356 (95% bound: 903–1911) cases were estimated to be attributable to HPV16/18 versus 1547 (95% bound: 1061–2124) cases attributable to HPV16/18/31/33/45/52/58. The estimated annual number of new vulvar cancer cases associated with the five new HPV types targeted by the nine-valent vaccine (HPV31/33/45/52/58) was thus 193 (95% bound: 71–401) (Table 2).

##### 3.1.1.3 Vaginal cancer

The estimated annual number of new vaginal cancer cases was 2171 (95% bound: 1676–2684). Of these cases, 1544 (95% bound: 1046–2107) are estimated to be attributable to HPV, assuming an overall HPV prevalence in vaginal cancer of 71.1% (95% CI: 63.2–78.1) in Europe [25]. The relative contribution of HPV16/18 and HPV16/18/31/33/45/52/58 was estimated to be 71.2% (95% CI: 61.8–79.6) and 85.6% (95% CI: 77.1–91.3), respectively (Table 1). Applying these values, 1099 (95% bound: 635–1685) cases were estimated to be attributable to HPV16/18 versus 1322 (95% bound: 799–1927) cases attributable to HPV16/18/31/33/45/52/58. Thus the estimated annual number of new vaginal cancers in Europe associated with the five new HPV types targeted by the nine-valent vaccine was 222 (95% bound: 69–483) (Table 2).

##### 3.1.1.4 Anal cancer

The estimated annual number of new anal cancer cases was 4562 (95% bound: 3875–5265) among women in Europe, irrespective of HPV status. Given the overall HPV prevalence in anal cancer of 87.6% (95% CI: 81.6–92.1) [19], 3996 cases (95% bound: 3151–4855) were estimated to be attributable to HPV. The relative contribution of HPV16/18 in HPV-positive anal cancers was estimated at 87.1% (95% CI: 80.7–92.1) and the relative contribution of HPV16/18/31/33/45/52/58 at 89.8% (95% CI: 83.8–94.2). After applying these values, 3481 (95% bound: 2533–4477) cases were estimated to be attributable to HPV16/18 versus 3589 (95% bound: 2632–4577) attributable to HPV16/18/31/33/45/52/58 (Table 1). Thus an additional 108 (95% bound: 13–356) cases were estimated to be attributable to the five new HPV types targeted by the nine-valent vaccine (Table 2).

#### 3.1.2 HPV-related precancerous lesions in women

##### 3.1.2.1 Cervical intraepithelial neoplasia grade 2 or worse

Age-standardized incidence rates of CIN2+ (including CIN2/3 and AIS) in the five countries for which data were available ranged between 138.8 (Norway) and 183.2 (Iceland) per 100,000 woman-years (Appendix A). Incidence was very low in the youngest age group (<20 years) in the four Nordic countries considered (there were no data available for this age group in France, where cervical cancer screening starts at age 25 years) [16]. However, in Iceland, where organized cervical cancer screening begins at 20 years of age, incidence rates in this age group were slightly higher, and the highest rates were observed from age 20 to 30 years. After the age of 50 years age-specific incidence rates in Iceland were the lowest of the four Nordic countries considered.

**Fig. 2:**
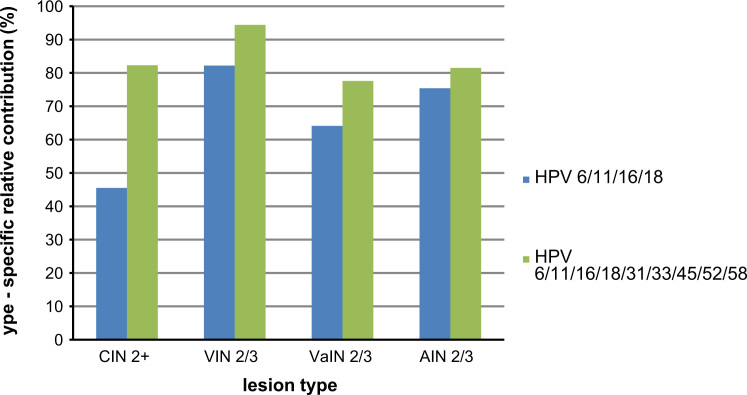
Relative contribution of HPV types 6/11/16/18 versus 6/11/16/18/31/33/45/52/58 in precancerous lesions. HPV: human papillomavirus; CIN: cervical intraepithelial neoplasia; CIN2+ includes CIN2/3 and AIS; VIN: vulvar intraepithelial neoplasia; VaIN: vaginal intraepithelial neoplasia; AIN: anal intraepithelial neoplasia.

All CIN is HPV-related, with HPV6/11/16/18 accounting for 23–25% of CIN1, 38.4–39% of CIN2, and 58% of CIN3. The HPV types targeted by the nine-valent vaccine account for 46–51% of CIN1, 71–74.3% of CIN2 and 85–90% of CIN3 (worldwide data) [17] (Table 3). Based on the age-specific incidence rates, the estimated annual number of new CIN2+ cases in women in Europe ranged between 267,350 and 510,609. 45.5% and 82.3% of these cases were estimated to be attributable to the HPV types targeted by the quadrivalent vaccine (HPV6/11/16/18) and nine-valent vaccine (HPV6/11/16/18/31/33/45/52/58), respectively [17 with additional information on European data kindly provided by the authors] (Table 3, (Fig. 2). After applying these values, 121,644 to 232,327 of the 267,350 to 510,609 new annual CIN2+ cases were estimated to be attributable to HPV6/11/16/18 versus 220,029 to 420,231 cases for HPV6/11/16/18/31/33/45/52/58. Therefore an additional 98,385 to 187,904 CIN2+ cases annually are estimated to be related to the five new HPV types included in the nine-valent vaccine (31/33/45/52/58) (Table 4).

**Table 3:** Overall HPV prevalence and the relative contribution^a^ of HPV6/11/16/18 and HPV6/11/16/18/31/33/45/52/58 by precancerous lesion.

Precancerous lesion	HPV prevalence %, (95% CI)	HPV6/11/16/18 attributable fraction HPV+ cases %, (95% CI)	HPV 6/11/16/18/31/33/45/52/58 attributable fraction among HPV+ cases %, (95% CI)	HPV 31/33/45/52/58 attributable fraction among HPV+ cases %	Reference
CIN 1^b^	100	23–25	51–46	+23–26	Joura et al. [17]
CIN 2^b^	100	38.4–39	71–74.3	+32–35.9	Joura et al. [17]
CIN 3^b^	100	58	85–90	+27–32.	Joura et al. [17]
CIN 2+^c^	100	45.5	82.3	+36.8	Joura et al. [17] with additional information kindly provided by the authors
VIN 2/3^c^	86.9 (82.6–90.4)	82.2 (77.2–86.6)	94.4 (91.0–96.9)	+12.2	de Sanjosé et al. [18]
VaIN 2/3^c^	95.8 (91.8–98.2)	64.1 (56.6–71.2)	77.6 (70.6–83.3)	+13.5	Alemany et al. [25]
AIN 2/3^c^	95.3 (84.2–99.4)	75.4 (59.4–87.4)	81.5 (66.4–91.9)	+6.1	Alemany et al. [19]

HPV: human papillomavirus; CIN: cervical intraepithelial neoplasia; VIN: vulvar intraepithelial neoplasia; VaIN: vaginal intraepithelial neoplasia; AIN: anal intraepithelial neoplasia; CI: confidence interval.

a Adjusted for multiple infections.

b Worldwide data.

c European data.

**Table 4:** Estimated annual number of new CIN2+, VIN 2/3, VaIN 2/3 and AIN 2/3 cases in women and men in Europe.

**Precancerous lesion**	***N*****of new precancerous lesions irrespective of HPV status**	***N*****of cases attributable to HPV**	***N*****of cases related to quadrivalent vaccine types (range)**	***N*****of cases related to nine-valent vaccine types (range)**	**Additional*****N*****of cases related to five new HPV types**
CIN 2+	267,350–510,609	267,350–510,609	121,644–232,327	220,029–420,231	98,385–187,904
VIN 2/3	13,886–27,592	12,067–23,977	9,919–19,709	11,391–22,635	1,472–2,925
VaIN 2/3	2,549–4,719	2,442–4,521	1,566–2,898	1,895–3,508	329–610
AIN 2/3 (F)	1,545	1,472	1,110	1,200	90
AIN 2/3 (M)	1,093	1,042	786	849	63
**Total (both sexes)**	**286,423–545,558**	**284,373–541,621**	**135,025–256,830**	**235,364–448,423**	**100,339–191,592**
**%**	**100**	**99**	**47**	**82**	**35**

HPV: human papillomavirus; CIN: cervical intraepithelial neoplasia; CIN 2+ includes CIN2/3 and AIS; VIN: vulvar intraepithelial neoplasia; VaIN: vaginal intraepithelial neoplasia; AIN: anal intraepithelial neoplasia; *N*: number.

##### 3.1.2.2 Vulvar intraepithelial neoplasia grades 2 and 3

The age-standardized incidence rates of VIN2/3 in the three Nordic countries for which data were available ranged between 4.8 (Norway) and 8.8 (Iceland) per 100,000 woman-years (Appendix B). Based on the age-specific incidence data from these countries, the estimated annual number of new VIN2/3 cases in Europe was estimated at between 13,886 and 27,592. Of these cases 86.9% (95% CI: 82.6–90.4) are believed to be HPV-related, with HPV16/18 accounting for 82.2% (95% CI: 77.2–86.6) and HPV16/18/31/33/45/52/58 accounting for 94.4% (95% CI: 91.0–96.9) of HPV-positive cases [18] (Table 3, Fig. 2). Based on these estimates, 12,067 to 23,977 of the VIN2/3 cases were estimated to be HPV-positive, with 9919 to 19,709 cases attributable to HPV16/18 versus 11,391 to 22, 635 attributable to HPV16/18/31/33/45/52/58. Thus the estimated annual number of new VIN2/3 cases associated with the five new HPV types included in the nine-valent vaccine ranged between 1472 and 2925 in women in Europe (Table 4).

##### 3.1.2.3 Vaginal intraepithelial neoplasia grades 2 and 3

Age-standardized incidence rates of VaIN2/3 in the three Nordic countries for which data were available ranged between 0.9 (Norway) and 1.3 (Iceland) per 100,000 woman-years (Appendix C). Based on age-specific incidence data from these countries, the estimated annual number of new VaIN2/3 cases in women in Europe ranged between 2549 and 4719. Of these cases, 95.8% (95% CI: 91.8–98.2) are expected to be HPV-related, with HPV16/18 accounting for 64.1% (95% CI: 56.6–71.2) and HPV16/18/31/33/45/52/58 accounting for 77.6% (95% CI: 70.6–83.3) of HPV-positive cases [25] (Table 3, Fig. 2). Based on these estimations, 2442 to 4521 of the 2549 to 4719 new annual VIN2/3 cases were expected to be HPV-positive, with 1566 to 2898 cases attributable to HPV16/18 versus 1895 to 3508 cases attributable to HPV16/18/31/33/45/52/58. The estimated annual number of new VaIN2/3 cases associated with the new HPV types included in the nine-valent vaccine (HPV31/33/45/52/58) thus ranged between 330 and 610 (Table 4).

##### 3.1.2.4 Anal intraepithelial neoplasia grades 2 and 3

Based on the age-standardized rate of 0.58 (per 100,000 person-years) for AIN2/3 in women [20], 1545 new AIN2/3 cases were estimated to occur each year in women in the 31 European countries. Of these cases, 95.3% (95% CI: 84.2–99.4%) are believed to be HPV-related [19]. Applying these values resulted in 1472 cases attributable to HPV, with 75.4% (95% CI: 59.4–87.4) and 81.5% (95% CI: 66.4–91.9) attributable to the HPV types targeted by the quadrivalent and the nine-valent vaccine, respectively (Table 3, Fig. 2), corresponding to 1110 and 1200 cases, respectively (Table 4).

#### 3.1.3 Genital warts

The most robust data on the incidence of genital warts come from Germany and the United Kingdom. Based on these studies, a lower incidence estimate of 142.0 per 100,000 woman-years [23] and an upper estimate of 191.1 per 100,000 woman-years [22] were extracted, and so the estimated annual number of new genital wart cases in women in Europe ranged between 378,141 and 508,893. Assuming an HPV6/11 prevalence in genital warts of 90% [2], between 340,327 and 458,003 of these cases were estimated to be attributable to HPV6/11 (Table 5).

**Table 5:** Estimated annual number of new genital wart cases in women and men in Europe.

	*N* of new annual cases (range)	*N* of new annual cases related to HPV6/11 (range)
Women	378,141–508,893	340,327–458,003
Men	375,467–426,425	337,921–383,782
**Total (both sexes)**	**753,608–935,318**	**678,248–841,785**

HPV: human papillomavirus; *N*: number.

### 3.2 Men

#### 3.2.1 HPV-related cancers

##### 3.2.1.1 Anal cancer

The estimated annual number of new anal cancers in men in Europe was estimated at 2729 (95% bound: 2195–3279), irrespective of HPV status. Given the overall HPV prevalence of 87.6% (95% CI: 81.6–92.1) in anal cancer [19], 2390 (95% bound: 1783–3025) of these cases were estimated to be attributable to HPV. The relative contribution of HPV16/18 in HPV-positive anal cancer has been estimated at 87.1% (95% CI: 80.7–92.1), with the relative contribution of HPV16/18/31/33/45/52/58 at 89.8% (95% CI: 83.8–94.2). After applying these values, 2082 (95% bound: 1431–2790) cases were estimated to be attributable to HPV16/18 versus 2147 (95% bound: 1487–2852) attributable to HPV16/18/31/33/45/52/58 (Table 1), thus an additional 65 (95% bound: 6–226) cases were estimated to be attributable to five new HPV types included in the nine-valent vaccine (Table 2).

#### 3.2.2 HPV-related precancerous lesions

##### 3.2.2.1 Anal intraepithelial neoplasia grades 2 and 3

Based on the age-standardized rate of AIN2/3 in men of 0.43 [20], 1093 new AIN2/3 cases were expected to occur each year in men in the 31 European countries and an 95.3% (95% CI: 84.2–99.4%) of AIN2/3 are HPV-related [19]. This rendered 1042 cases, with 75.4% (95% CI: 59.4–87.4) and 81.5% (95% CI: 66.4–91.9) attributable to the HPV types targeted by the quadrivalent and the nine-valent vaccine, and corresponded to an estimated annual number of 786 and 849 cases, respectively (Table 3, Table 4).

#### 3.2.3 Genital warts

The most robust data on the incidence of genital warts come from Germany [22] and the United Kingdom [23]: Based on these studies, a lower incidence estimate of 147.66 per 100,000 man-years and an upper estimate of 167.7 per 100,000 man-years were extracted, and so the estimated annual number of new genital wart cases in men in Europe ranged between 375,467 and 426,425. Assuming an HPV6/11 prevalence in genital warts of 90% [2], between 337,921 and 383,782 of these cases were estimated to be attributable to HPV6/11 (Table 5).

## 4 Discussion

**Fig. 3:**
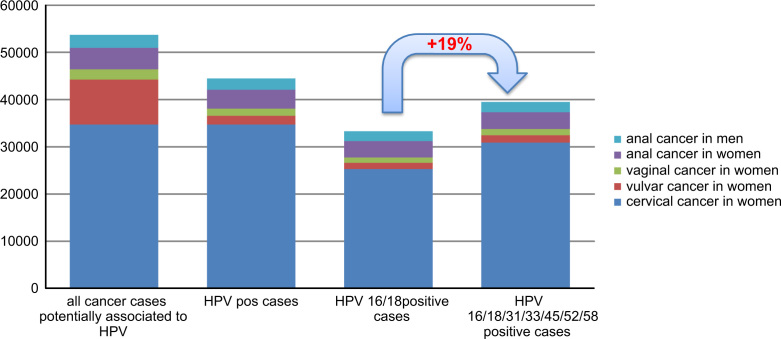
Estimated annual number of new HPV-related cancer cases in women in Europe associated with the HPV types targeted by the quadrivalent and the nine-valent vaccine. HPV: human papillomavirus.

Our estimates demonstrate the high burden of cancer and precancerous lesions associated with the HPV types targeted by the new nine-valent HPV vaccine (HPV6/11/16/18/31/33/45/52/58) that are expected to occur every year in women and men in Europe. Overall, 39,494 (95% bound: 34,787 to 44,372) of the 53,714 (95% bound: 49,296 to 58,165) new HPV-related cancers of the cervix, vulva, vagina, and anus in women and men in Europe are expected to be associated with the high-risk HPV types included in the nine-valent vaccine, versus 33,285 (95% bound: 28,818 to 38,055) associated with high-risk HPV16/18. This represents an additional 6210 cancer cases (95% bound: 4892–7981), corresponding to a relative increase of 19% (Fig. 3).

The proportion of cervical cancer attributable to the seven high-risk HPV types targeted by the nine-valent vaccine is similar worldwide. The combined relative contribution of these seven high-risk types in Europe was 89.0% (95% CI: 87.5–90.3) compared to worldwide values of 89.4% (95% CI: 88.8–90.1), which range from 84.6% (95% CI: 81.9–87.1) in Central America to 95.5% (95% CI: 91.2–98.2) in North America [26].

**Fig. 4:**
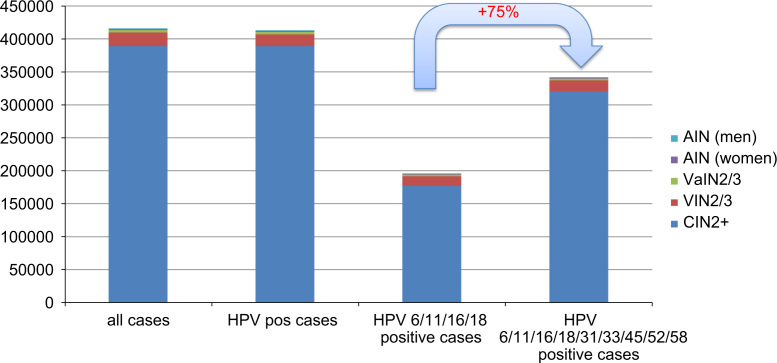
Estimated annual number of new HPV-related precancerous lesions of the cervix, vulva, vagina and anus associated with the HPV types targeted by the quadrivalent and the nine-valent vaccine in women in Europe. HPV: human papillomavirus; CIN: cervical intraepithelial neoplasia; CIN2+ includes CIN2/3 and AIS; VIN: vulvar intraepithelial neoplasia; VaIN: vaginal intraepithelial neoplasia; AIN: anal intraepithelial neoplasia.

Furthermore, the estimated annual number of new cases of precancerous lesions occurring in women and men in Europe (CIN2/3+AIS, VIN2/3, VaIN2/3 and AIN2/3) was 286,423 to 545,558, with 284,373 to 541,621 HPV-positive cases. Of these, 235,364 to 448,423 cases are associated with HPV6/11/16/18/31/33/45/52/58 versus 135,025 to 256,830 associated with HPV6/11/16/18. The five new HPV types 31/33/45/52/58 were thus associated with an additional 100,339 to 191,592 cases per year, corresponding to a relative increase of 75% (Table 4, (Fig. 4). We did not estimate the expected annual number of new low-grade precancerous lesions of the cervix (CIN1). Indeed, no robust incidence data are available on CIN1 as these cases are not usually reported to cancer registries, and a high amount of this cases regress spontaneously. Thus records of CIN1 incidence very much depend on the national screening system and cannot be extrapolated. Even if CIN1 is not currently considered a precancerous lesion, the potential psychological and economic impact (due to medical follow-up) on affected women is very important. The additional number of CIN1 cases related to the five new HPV types 31/33/45/52/58 is expected to be very high, as worldwide 46–51% of CIN1 are related to the HPV types targeted by the nine-valent vaccine compared to 23–25% for the quadrivalent vaccine types [17].

As previously mentioned, there are other cancers known to be associated with HPV, including penile cancer in men and a subset of head and neck cancers in both sexes. The currently licensed HPV vaccines don’t have an indication for cancers at these anatomical sites and up to now efficacy of the vaccines against these cancers has not been demonstrated. However, it can be expected that future studies based on routine use of the vaccines may demonstrate their efficacy against head and neck cancers and penile cancers.

Based on the above described methodology we estimated the overall burden of head and neck cancers in women and men and penile cancer in men in Europe. 17,676 new head and neck cancer cases were estimated to occur every year in women, with 2089 and 1915 related to second and first generation vaccine types, respectively. For men the number of head and neck cancers was 80,062, of which 7911 were related to second and 7144 cases to first generation vaccine types. The burden of penile cancer was 4096 this region with 1070 and 932 cases related to second and first generation vaccines, respectively (work in progress). These data are preliminary, as the key studies estimating the prevalence of HPV at these sites, and more specifically the attributable fraction of the seven high-risk HPV types targeted by the nine-valent vaccine, are still ongoing. The data currently available in the literature are very heterogeneous, as only a subset of head and neck cancers is HPV-related, and many other risk factors exist, like alcohol and tobacco consumption, which differ from region to region. Moreover, differences in the results of HPV prevalence between studies might be due to contamination and the variability of HPV detection methods. Studies considering more accurate biomarkers of oncogenic HPV activity, such as E6/E7 mRNA or p16INK4a in addition to HPV DNA prevalence, are still ongoing.

Recurrent respiratory papillomatosis is also highly associated with HPV. It is a very rare disease, which is difficult to treat and has high recurrence rates. However, incidence data in Europe are scarce [27], and thus it was not possible to estimate the number of new annual cases in Europe. Low-risk HPV6/11 are prevalent in virtually all cases [27], [28]. and no additional burden is expected to be associated with the five new HPV types.

### 4.1 Strengths and limitations

A short-term prediction method was used to estimate the number of new cancer cases in 2013 from the most recent data collected from 2003 to 2007. Therefore these estimates were accurate only if the incidence rates of the cancers under study remained stable over time. In the case of increasing incidence, they would slightly underestimate the expected number of cases, and the opposite would be true in the case of decreasing incidence.

As mentioned above, the CI5 database contains national cancer incidence rates for 19 European countries. Eight of the countries included in this report had only regional incidence rates available, which were extrapolated to the entire country. Although we assessed the geographical coverage and distribution of these regional registries, other factors could vary and influence regional incidence rates. For the remaining four countries no robust regional or national data were available. We thus extrapolated the mean incidence data from surrounding cancer registries to these countries, but we had no means to check the robustness of this method. Therefore the results should thus be interpreted with particular caution.

For some cancer sites additional tests such as p16 or mRNA had been performed to confirm causality in the presence of HPV DNA (lack of data availability). Indeed, the mere presence of HPV is insufficient to prove causation, as the infection may be transient and not related to the carcinogenic process. Therefore our application of previously published HPV prevalence to an estimated number of new cancer cases may have yielded an overestimation of cases attributable to HPV.

Our calculations were based on the relative contribution estimates extracted from one study per cancer site. However, for each cancer site we selected the study with the most robust design and a large sample size, and that provided individual data allowing weighting for multiple infections. We used only European data and, when available, adjusted data rather than crude data. Sex-specific data for HPV prevalence in anal cancers were available, but we decided not to use it, as sample size by sex was not sufficient to obtain meaningful, precise estimates. In any case, there were no sizeable differences between the data for both sexes. It was not possible to compare the extracted data to results from meta-analyses, as to our knowledge no meta-analysis contains information about multiple infections in precancerous lesions, and thus the relative contribution of individual HPV types, taking multiple infection into account (i.e., to avoid double counting) could not be estimated. A literature search that we performed in parallel showed large differences in the prevalence of the different HPV types between studies and regions. However, it is not clear if these differences are due to real differences in the distribution of HPV types among European women, or to differences in HPV detection methods. Furthermore, most of the published studies contained no information about individual multiple infections or had low sample sizes. The calculation of a robust relative contribution of specific types was thus not possible based on these data, as it would have provided less meaningful results.

The method used to calculate the estimated annual number of new CIN cases also has some limitations. For CIN2+ we based our estimations on data extracted from one published epidemiological study covering four Nordic countries and one French study (unpublished). To calculate the estimated annual number of new CIN2+ cases in women in Europe we extrapolated the lower and the upper mean value of each age group to the population of each included European country. However, the incidence of CIN may vary throughout Europe, not only due to regional differences in the prevalence of HPV, but also due to different screening and prevention methods.

In Iceland for example, screening starts at age 20, with a 2-year interval until age 69. This might explain the high incidence rates observed in the youngest age groups and lower incidence rates in the age group over 50 in Iceland compared to the other four countries (personal communication from Kristján Oddsson).

We used data from countries with well-organized screening, thus reflecting the real high burden of precancerous lesions. By extrapolating these data to countries without or with less organized screening, we overestimated the detected burden in those countries. However these lesions do exist, even if not detected, and have a high potential to develop to cancer if not recognized and treated: 5% of CIN2 and more than 12% of CIN3 progress to invasive cancer [29]. Mc Credie et al. found that the cumulative 30 year incidence of invasive cancer in women with CIN3 was 30% and increased to 50% in women with persistent CIN3 [30]. Therefore we consider that the estimation of the detectable burden (referring to a real but not recognized burden in countries without or with limited screening) is of high value.

Another limitation is that our estimations of CIN2+ were based on estimates of the relative contribution of HPV observed in a population of young women (aged 16 to 26 years), who may have more multiple infections than older women. The burden of precancerous lesions is most important in the younger age group, and in the absence of age-specific relative contribution estimates, it seemed appropriate to use the data for all age groups combined.

Finally, it has to be considered that all results on the incidence of cancers and cervical precancerous lesions in this study have been estimated in a post-screening context.

The reporting of VIN2/3 and VaIN2/3 might not be exhaustive as there is no dedicated screening program, thus the reported burden is probably underestimated. Differences in the incidence of these lesions were observed between the different countries and it remains unclear to what extent the observed discrepancies can be explained by the completeness of registries, differences in clinical practice and management, or different background risk. Compared to a study from the Netherlands [31], much higher rates of VIN2/3 were reported in the study from Nygard et al. [16]. To our knowledge the data that we used for our estimation are the best data available in Europe as there is no organized screening and mandatory notification for these precancerous lesions. However, the results have to be considered with caution. Additional studies in the future are necessary to give a better insight on the real burden of these lesions.

## 5 Conclusions

A total number of 53,714 new cases (95% bound: 49,296–58,165) of cancers of the cervix, vulva, vagina in women, and anus in women and men, were estimated to occur in Europe every year, of which 44,480 (95% bound: 40,294–48,795) were estimated to be HPV-positive. Of the latter, 89%, i.e., 39,494 cases (95% bound: 34,787–44,372) were estimated to be related to HPV types 16/18/31/33/45/52/58 versus 75% to HPV16/18, corresponding to 33,285 cases (95% bound: 28,818–38,055).

Additionally, the estimated annual burden of CIN2+, VIN2/3, VaIN2/3 in women, and AIN2/3 in women and men ranged between 286,423 and 545,558 new cases per year, of which 82% were related to HPV6/11/16/18/31/33/45/52/58 (i.e., 235,364 to 448,423 cases versus 47% related to HPV6/11/16/18), corresponding to 135,025 to 256,830 cases.

In addition to cancers and precancerous lesions, between 753,608 and 935,318 new annual genital warts cases were estimated to occur in women and men in Europe, of which 90% (between 678,248 and 841,785 cases) were estimated to be related to HPV6/11.

The relative increase in the number of new cancers attributable to HPV16/18/31/33/45/52/58 compared to HPV16/18 was 19%. For precancerous lesions this increase was 75% when comparing HPV6/11/16/18/31/33/45/52/58 and HPV6/11/16/18

These data demonstrate how the large public health impact in the prevention of cancer that was achieved by the first generation HPV vaccines could be further increased by the second generation nine-valent HPV vaccine, due to additional cancer prevention and notably the prevention of precancerous lesion in women.

## Competing interests

SH and GDF are employees of Sanofi Pasteur MSD; JJB has been a member of the scientific advisory board of MSD Sanofi-Pasteur and GSK, has received speaker’s fees and travel grants from MSD Sanofi-Pasteur and GSK, and research supports from MSD Sanofi-Pasteur; FS was employee of Sanofi Pasteur MSD at the time of study initiation; LA received occasional travel grants to attend scientific meetings from MSD and Sanofi Pasteur MSD; SDS received travel grants from MSD, GSK and Qiagen and unrestricted research grants through ICO from Merck & Co. Inc. and Glaxo Smith Kline.; XC has received speaker honoraria, travel grants to attend scientific meetings from SPMSD, and research funding through ICO to undertake HPV studies from Merck & Co. Inc., Glaxo Smith Kline, and Sanofi Pasteur MSD.

## Authors’ contributions

SH contributed to the study design, literature research, data analysis, interpretation of findings and drafting of the manuscript. JJB contributed to the data collection and interpretation of findings. GDF contributed to the study design, and interpretation of findings. FS contributed to the study design, data-analysis, interpretation of findings, and critical editing of the manuscript. LA contributed to the interpretation of findings. SDS contributed to data collection, interpretation of findings, and critical editing of the manuscript. XC contributed to the study design, data collection, interpretation of findings, and critical editing of the manuscript. All authors critically reviewed the manuscript and approved the final version.
