# Supplementary material for: Estimation of the epidemiological burden of HPV-related anogenital cancers, precancerous lesions, and genital warts in women and men in Europe: Potential additional benefit of a nine-valent second generation HPV vaccine compared to first generation HPV vaccines
Source: Papillomavirus Res. 2015 Jun 16;1:90–100. doi: 10.1016/j.pvr.2015.06.003 (PMC5886848; doi:10.1016/j.pvr.2015.06.003)
Supplement: Supplementary file 1 — Supplementary material [file mmc1.docx]

**Annex 1 - Estimated mean annual number of new HPV-related cancer cases in women and men per European country**

**A Cervical cancer**

| **Country** | **N of new cancers irrespective of HPV status (95% CI)** | **N of new cancers attributable to HPV (95% CI)^1^** | **N of new cancers attributable to HPV16/18 (95% CI)^2^** | **N of HPV16/18/31/33/45/52/58+ cancers**  **(95% CI)^3^** | **N of cases attribu-table to additional types Gardasil9 vs Gardasil** |
| --- | --- | --- | --- | --- | --- |
| Austria | 626  (577 - 675) | 626  (577 - 675) | 456  (414 - 498) | 557  (511 - 603) | 101  (81 - 121) |
| Belgium | 693  (641 - 745) | 693  (641 - 745) | 504  (460 - 548) | 617  (568 - 666) | 112  (91 - 133) |
| Bulgaria | 1094  (1029 - 1159) | 1094  (1029 - 1159) | 796  (741 - 851) | 974  (913 - 1035) | 177  (151 - 203) |
| Croatia | 345  (309 - 381) | 345  (309 - 381) | 251  (220 - 282) | 307  (273 - 341) | 56  (41 - 71) |
| Cyprus | 28  (18 - 38) | 28  (18 - 38) | 21  (12 - 30) | 25  (15 - 35) | 5  (2 - 12) |
| Czech Republic | 1095  (1030 - 1160) | 1095  (1030 - 1160) | 797  (742 - 852) | 975  (914 - 1036) | 177  (151 - 203) |
| Denmark | 397  (358 - 436) | 397  (358 - 436) | 289  (256 - 322) | 353  (316 - 390) | 64  (48 - 80) |
| Estonia | 164  (139 - 189) | 164  (139 - 189) | 119  (98 - 140) | 146  (122 - 170) | 27  (17 - 37) |
| Finland | 164  (139 - 189) | 164  (139 - 189) | 119  (98 - 140) | 146  (122 - 170) | 26  (16 - 36) |
| France | 3400  (3286 - 3514) | 3400  (3286 - 3514) | 2475  (2377 - 2573) | 3026  (2918 - 3134) | 551  (505 - 597) |
| Germany | 5299  (5156 - 5442) | 5299  (5156 - 5442) | 3858  (3736 - 3980) | 4716  (4581 - 4851) | 858  (801 - 915) |
| Greece | 1488  (1412 - 1564) | 1488  (1412 - 1564) | 1084  (1019 - 1149) | 1325  (1254 - 1396) | 241  (211 - 271) |
| Hungary | 985  (923 - 1047) | 985  (923 - 1047) | 717  (665 - 769) | 877  (819 - 935) | 160  (135 - 185) |
| Iceland | 15  (8 - 25) | 15  (8 - 25) | 11  (5 - 20) | 13  (7 - 22) | 2  (0 - 7) |
| Ireland | 278  (245 - 311) | 278  (245 - 311) | 202  (174 - 230) | 247  (216 - 278) | 45  (32 - 58) |
| Italy | 2645  (2544 - 2746) | 2645  (2544 - 2746) | 1926  (1840 - 2012) | 2354  (2259 - 2449) | 429  (388 - 470) |
| Latvia | 214  (185 - 243) | 214  (185 - 243) | 156  (132 - 180) | 191  (164 - 218) | 35  (23 - 47) |
| Lithuania | 489  (446 - 532) | 489  (446 - 532) | 356  (319 - 393) | 436  (395 - 477) | 79  (62 - 96) |
| Luxem-bourg | 30  (19 - 41) | 30  (19 - 41) | 22  (13 - 31) | 27  (17 - 37) | 5  (2 - 12) |
| Malta | 12  (6 - 21) | 12  (6 - 21) | 8  (3 - 16) | 10  (5 - 18) | 2  (0 - 7) |
| Norway | 313  (278 - 348) | 313  (278 - 348) | 228  (198 - 258) | 279  (246 - 312) | 51  (37 - 65) |
| Poland | 3691  (3572 - 3810) | 3691  (3572 - 3810) | 2687  (2585 - 2789) | 3285  (3173 - 3397) | 598  (550 - 646) |
| Portugal | 1009  (947 - 1071) | 1009  (947 - 1071) | 734  (681 - 787) | 898  (839 - 957) | 163  (138 - 188) |
| Romania | 2691  (2589 - 2793) | 2691  (2589 - 2793) | 1959  (1872 - 2046) | 2395  (2299 - 2491) | 436  (395 - 477) |
| Slovakia | 622  (573 - 671) | 622  (573 - 671) | 453  (411 - 495) | 553  (507 - 599) | 101  (81 - 121) |
| Slovenia | 180  (154 - 206) | 180  (154 - 206) | 131  (109 - 153) | 160  (135 - 185) | 29  (18 - 40) |
| Spain | 2308  (2214 - 2402) | 2308  (2214 - 2402) | 1680  (1600 - 1760) | 2054  (1965 - 2143) | 374  (336 - 412) |
| Sweden | 466  (424 - 508) | 466  (424 - 508) | 339  (303 - 375) | 415  (375 - 455) | 76  (59 - 93) |
| Switzer-land | 261  (229 - 293) | 261  (229 - 293) | 190  (163 - 217) | 232  (202 - 262) | 42  (29 - 55) |
| The Nether-lands | 702  (650 - 754) | 702  (650 - 754) | 511  (467 - 555) | 625  (576 - 674) | 114  (93 - 135) |
| United Kingdom | 3004  (2897 - 3111) | 3004  (2897 - 3111) | 2187  (2095 - 2279) | 2674  (2573 - 2775) | 487  (444 - 530) |

^1^ HPV prevalence: 100%, ^2^ HPV 16/18 attributable fraction among HPV+ cases: 72.8% (70.8–74.7) , ^3^ HPV 31/33/45/52/558 attributable fraction among HPV+ cases: 89.0% (87.5–90.3) (ref: de Sanjosé et al [10])

**B Vulvar cancer**

| **Country** | **N of new cancers irrespective of HPV status (95% CI)** | **N of new cancers attributable to HPV (95% CI)^4^** | **N of new cancers attributable to HPV16/18 (95% CI)^5^** | **N of HPV16/18/31/33/45/ 52/58+ cancers**  **(95% CI)^6^** | **N of cases attributable to additional types Gardasil9 vs Gardasil** |
| --- | --- | --- | --- | --- | --- |
| Austria | 134  (111 - 157) | 26  (16 - 36) | 19  (11 - 30) | 22  (13 - 31) | 3  (1 - 9) |
| Belgium | 196  (169 - 223) | 38  (26 - 50) | 28  (18 - 38) | 32  (21 - 43) | 4  (1 - 10) |
| Bulgaria | 128  (106 - 150) | 25  (15 - 35) | 18  (11 - 28) | 21  (12 - 30) | 3  (1 - 9) |
| Croatia | 75  (58 - 92) | 15  (8 - 25) | 11  (5 - 20) | 12  (6 - 21) | 2  (0 - 7) |
| Cyprus | 9  (4 - 17) | 2  (0 - 7) | 1  (0 - 6) | 1  (0 - 6) | 0  (0 - 4) |
| Czech Republic | 224  (195 - 253) | 43  (30 - 56) | 32  (21 - 43) | 36  (24 - 48) | 4  (1 - 10) |
| Denmark | 101  (81 - 121) | 20  (11 - 29) | 14  (8 - 23) | 16  (9 - 26) | 2  (0 - 7) |
| Estonia | 29  (18 - 40) | 6  (2 - 13) | 4  (1 - 10) | 5  (2 - 12) | 1  (0 - 6) |
| Finland | 84  (66 - 102) | 16  (9 - 26) | 12  (6 - 21) | 14  (8 - 23) | 2  (0 - 7) |
| France | 753  (699 - 807) | 145  (121 - 169) | 107  (87 - 127) | 122  (100 - 144) | 15  (8 - 25) |
| Germany | 2485  (2387 - 2583) | 480  (437 - 523) | 353  (316 - 390) | 403  (364 - 442) | 50  (36 - 64) |
| Greece | 189  (162 - 216) | 37  (25 - 49) | 27  (17 - 37) | 31  (20 - 42) | 4  (1 - 10) |
| Hungary | 194  (167 - 221) | 38  (26 - 50) | 28  (18 - 38) | 32  (21 - 43) | 4  (1 - 10) |
| Iceland | 3  (1 - 9) | 0  (0 - 4) | 0  (0 - 4) | 0  (0 - 4) | 0  (0 - 4) |
| Ireland | 52  (38 - 66) | 10  (5 - 18) | 7  (3 - 14) | 8  (3 - 16) | 1  (0 - 6) |
| Italy | 1133  (1067 - 1199) | 219  (190 - 248) | 161  (136 - 186) | 184  (157 - 211) | 23  (14 - 32) |
| Latvia | 42  (29 - 55) | 8  (3 - 16) | 6  (2 - 13) | 7  (3 - 14) | 1  (0 - 6) |
| Lithuania | 59  (44 - 74) | 11  (5 - 20) | 8  (3 - 16) | 10  (5 - 18) | 1  (0 - 6) |
| Luxem-bourg | 10  (5 - 18) | 2  (0 - 7) | 1  (0 - 6) | 2  (0 - 7) | 0  (0 - 4) |
| Malta | 9  (4 - 17) | 2  (0 - 7) | 1  (0 - 6) | 1  (0 - 6) | 0  (0 - 4) |
| Norway | 97  (78 - 116) | 19  (11 - 30) | 14  (8 - 23) | 16  (9 - 26) | 2  (0 - 7) |
| Poland | 500  (456 - 544) | 96  (77 - 115) | 71  (54 - 88) | 81  (63 - 99) | 10  (5 - 18) |
| Portugal | 109  (89 - 129) | 21  (12 - 30) | 15  (8 - 25) | 18  (11 - 28) | 2 (0 - 7) |
| Romania | 325  (290 - 360) | 63  (47 - 79) | 46  (33 - 59) | 53  (39 - 67) | 7  (3 - 14) |
| Slovakia | 79  (62 - 96) | 15  (8 - 25) | 11  (5 - 20) | 13  (7 - 22) | 2  (0 - 7) |
| Slovenia | 42  (29 - 55) | 8  (3 - 16) | 6  (2 - 13) | 7  (3 - 14) | 1  (0 - 6) |
| Spain | 706  (654 - 758) | 136  (113 - 159) | 100  (80 - 120) | 114  (93 - 135) | 14 (  8 - 23) |
| Sweden | 169  (144 - 194) | 33  (22 - 44) | 24  (14 - 34) | 27  (17 - 37) | 3  (1 - 9) |
| Switzer-land | 127  (105 - 149) | 24  (14 - 34) | 18  (11 - 28) | 21  (12 - 30) | 3  (1 - 9) |
| The Nether-lands | 318  (283 - 353) | 61  (46 - 76) | 45  (32 - 58) | 52  (38 - 66) | 6  (2 - 13) |
| United Kingdom | 1162  (1095 - 1229) | 224  (195 - 253) | 165  (140 - 190) | 188  (161 - 215) | 23  (14 - 32) |

^4^ HPV prevalence: 19.3% (16.7–22.0), ^5^ HPV 16/18 attributable fraction among HPV+ cases: 73.6% (66.4–79.9), ^6^ HPV 31/33/45/52/558 attributable fraction among HPV+ cases: 84.0% (77.6–89.0) (ref: de Sanjosé et al [18])

**C Vaginal cancer**

| **Country** | **N of new cancers irrespective of HPV status (95% CI)** | **N of new cancers attributable to HPV (95% CI)^7^** | **N of new cancers attributable to HPV16/18 (95% CI)^8^** | **N of HPV16/18/31/33/45/52/58+ cancers**  **(95% CI)^9^** | **N of cases attributable to additional types Gardasil9 vs Gardasil** |
| --- | --- | --- | --- | --- | --- |
| Austria | 61  (46 - 76) | 43  (30 - 56) | 31  (20 - 42) | 37  (25 - 49) | 6  (2 - 13) |
| Belgium | 54  (40 - 68) | 38  (26 - 50) | 27  (17 - 37) | 33  (22 - 44) | 6  (2 - 13) |
| Bulgaria | 29  (18 - 40) | 20  (11 - 29) | 15  (8 - 25) | 18  (11 - 28) | 3  (1 - 9) |
| Croatia | 16  (9 - 26) | 12  (6 - 21) | 8  (3 - 16) | 10  (5 - 18) | 2  (0 - 7) |
| Cyprus | 1  (0 - 6) | 1  (0 - 6) | 0  (0 - 4) | 0  (0 - 4) | 0  (0 - 4) |
| Czech Republic | 53  (39 - 67) | 38  (26 - 50) | 27  (17 - 37) | 32  (21 - 43) | 5  (2 - 12) |
| Denmark | 26  (16 - 36) | 19  (11 - 30) | 13  (7 - 22) | 16  (9 - 26) | 3  (1 - 9) |
| Estonia | 6  (2 - 13) | 4  (1 - 10) | 3  (1 - 9) | 4  (1 - 10) | 1  (0 - 6) |
| Finland | 22  (13 - 31) | 15  (8 - 25) | 11  (5 - 20) | 13  (7 - 22) | 2  (0 - 7) |
| France | 232  (202 - 262) | 165  (140 - 190) | 117  (96 - 138) | 141  (118 - 164) | 24  (14 - 34) |
| Germany | 477  (434 - 520) | 339  (303 - 375) | 242  (212 - 272) | 290  (257 - 323) | 49  (35 - 63) |
| Greece | 44  (31 - 57) | 31  (20 - 42) | 22  (13 - 31) | 27  (17 - 37) | 4  (1 - 10) |
| Hungary | 49  (35 - 63) | 35  (23 - 47) | 25  (15 - 35) | 30  (19 - 41) | 5  (2 - 12) |
| Iceland | 0  (0 - 4) | 0  (0 - 4) | 0  (0 - 4) | 0  (0 - 4) | 0  (0 - 4) |
| Ireland | 14  (8 - 23) | 10  (5 - 18) | 7  (3 - 14) | 8  (3 - 16) | 1  (0 - 6) |
| Italy | 240  (210 - 270) | 171  (145 - 197) | 121  (99 - 143) | 146  (122 - 170) | 25  (15 - 35) |
| Latvia | 9  (4 - 17) | 6  (2 - 13) | 4  (1 - 10) | 5  (2 - 12) | 1  (0 - 6) |
| Lithuania | 11  (5 - 20) | 8  (3 - 16) | 6  (2 - 13) | 7  (3 - 14) | 1  (0 - 6) |
| Luxem-bourg | 2  (0 - 7) | 1  (0 - 6) | 1  (0 - 6) | 1  (0 - 6) | 0  (0 - 4) |
| Malta | 1  (0 - 6) | 1  (0 - 6) | 1  (0 - 6) | 1  (0 - 6) | 0  (0 - 4) |
| Norway | 17  (10 - 27) | 12  (6 - 21) | 9  (4 - 17) | 10  (5 - 18) | 2  (0 - 7) |
| Poland | 114  (93 - 135) | 81  (63 - 99) | 58  (43 - 73) | 70  (54 - 86) | 12  (6 - 21) |
| Portugal | 73  (56 - 90) | 52  (38 - 66) | 37  (25 - 49) | 44  (31 - 57) | 7  (3 - 14) |
| Romania | 78  (61 - 95) | 55  (40 - 70) | 39  (27 - 51) | 47  (34 - 60) | 8  (3 - 16) |
| Slovakia | 21  (12 - 30) | 15  (8 - 25) | 11  (5 - 20) | 13  (7 - 22) | 2  (0 - 7) |
| Slovenia | 8  (3 - 16) | 6  (2 - 13) | 4  (1 - 10) | 5  (2 - 12) | 1  (0 - 6) |
| Spain | 121  (99 - 143) | 86  (68 - 104) | 61  (46 - 76) | 73  (56 - 90) | 12  (6 - 21) |
| Sweden | 43  (30 - 56) | 31  (20 - 42) | 22  (13 - 31) | 26  (16 - 36) | 4  (1 - 10) |
| Switzer-land | 31  (20 - 42) | 22  (13 - 31) | 16  (9 - 26) | 19  (11 - 30) | 3  (1 - 9) |
| The Nether-lands | 56  (41 - 71) | 40  (28 - 52) | 28  (18 - 38) | 34  (23 - 45) | 6  (2 - 13) |
| United Kingdom | 261  (229 - 293) | 186  (159 - 213) | 132  (109 - 155) | 159  (134 - 184) | 27  (17 - 37) |

^7^ HPV prevalence: 71.1% (63.2–78.1), ^8^ HPV 16/18 attributable fraction among HPV+ cases: 71.2% (61.8–79.6), ^9^ HPV 31/33/45/52/558 attributable fraction among HPV+ cases: 85.6% (77.1–91.3) (ref: Alemany et al [25])

**D Anal cancer**

| **Country** | **Sex** | **N of new cancers irrespective of HPV status (95% CI)** | **N of new cancers attributable to HPV (95% CI)^10^** | **N of new cancers attributable to HPV16/18 (95% CI)^11^** | **N of HPV16/18/31/33/45/52/58+ cancers (95% CI)^12^** | **N of cases attributable to additional types Gardasil9 vs Gardasil** |
| --- | --- | --- | --- | --- | --- | --- |
| Austria | Women | 97  (78 - 116) | 85  (67 - 103) | 74  (57 - 91) | 76  (59 - 93) | 2  (0 - 7) |
|  | Men | 38  (26 - 50) | 33  (22 - 44) | 29  (18 - 40) | 30  (19 - 41) | 1  (0 - 6) |
|  | Both sexes | 135  (112 - 158) | 118  (97 - 139) | 103  (83 - 123) | 106  (86 - 126) | 3  (1 - 9) |
| Belgium | Women | 86  (68 - 104) | 75  (58 - 92) | 66  (50 - 82) | 68  (52 - 84) | 2  (0 - 7) |
|  | Men | 58  (43 - 73) | 51  (37 - 65) | 44  (31 - 57) | 46  (33 - 59) | 1  (0 - 6) |
|  | Both sexes | 144  (120 - 168) | 126  (104 - 148) | 110  (89 - 131) | 113  (92 - 134) | 3  (1 - 9) |
| Bulgaria | Women | 26  (16 - 36) | 22  (13 - 31) | 20  (11 - 29) | 20  (11 - 29) | 1  (0 - 6) |
|  | Men | 34  (23 - 45) | 30  (19 - 41) | 26  (16 - 36) | 27  (17 - 37) | 1  (0 - 6) |
|  | Both sexes | 59  (44 - 74) | 52  (38 - 66) | 45  (32 - 58) | 47  (34 - 60) | 1  (0 - 6) |
| Croatia | Women | 16  (9 - 26) | 14  (8 - 23) | 12  (6 - 21) | 12  (6 - 21) | 0  (0 - 4) |
|  | Men | 9  (4 - 17) | 8  (3 - 16) | 7  (3 - 14) | 7  (3 - 14) | 0  (0 - 4) |
|  | Both sexes | 24  (14 - 34) | 21  (12 - 30) | 19  (11 - 30) | 19  (11 - 30) | 1  (0 - 6) |
| Cyprus | Women | 3  (1 - 9) | 3  (1 - 9) | 2  (0 - 7) | 2  (0 - 7) | 0  (0 - 4) |
|  | Men | 2  (0 - 7) | 2  (0 - 7) | 2  (0 - 7) | 2  (0 - 7) | 0  (0 - 4) |
|  | Both sexes | 5  (2 - 12) | 4  (1 - 10) | 4  (1 - 10) | 4  (1 - 10) | 0  (0 - 4) |
| Czech Republic | Women | 76  (59 - 93) | 67  (51 - 83) | 58  (43 - 73) | 60  (45 - 75) | 2  (0 - 7) |
|  | Men | 45  (32 - 58) | 39  (27 - 51) | 34  (23 - 45) | 35  (23 - 47) | 1  (0 - 6) |
|  | Both sexes | 121  (99 - 143) | 106  (86 - 126) | 92  (73 - 111) | 95  (76 - 114) | 3  (1 - 9) |
| Denmark | Women | 72  (55 - 89) | 63  (47 - 79) | 55  (40 - 70) | 56  (41 - 71) | 2  (0 - 7) |
|  | Men | 35  (23 - 47) | 30  (19 - 41) | 26  (16 - 36) | 27  (17 - 37) | 1  (0 - 6) |
|  | Both sexes | 106  (86 - 126) | 93  (74 - 112) | 81  (63 - 99) | 84  (66 - 102) | 3  (1 - 9) |
| Estonia | Women | 10  (5 - 18) | 9  (4 - 17) | 8  (3 - 16) | 8  (3 - 16) | 0  (0 - 4) |
|  | Men | 4  (1 - 10) | 3  (1 - 9) | 3  (1 - 9) | 3  (1 - 9) | 0  (0 - 4) |
|  | Both sexes | 14  (8 - 23) | 12  (6 - 21) | 11  (5 - 20) | 11  (5 - 20) | 0  (0 - 4) |
| Finland | Women | 25  (15 - 35) | 22  (13 - 31) | 19  (11 - 30) | 20  (11 - 29) | 1  (0 - 6) |
|  | Men | 15  (8 - 25) | 13  (7 - 22) | 12  (6 - 21) | 12  (6 - 21) | 0  (0 - 4) |
|  | Both sexes | 40  (28 - 52) | 35  (23 - 47) | 30  (19 - 41) | 31  (20 - 42) | 1  (0 - 6) |
| France | Women | 804  (748 - 860) | 705  (653 - 757) | 614  (565 - 663) | 633  (584 - 682) | 19  (11 - 30) |
|  | Men | 313  (278 - 348) | 274  (242 - 306) | 238  (208 - 268) | 246  (215 - 277) | 7  (3 - 14) |
|  | Both sexes | 1117  (1051 - 1183) | 979  (918 - 1040) | 852  (795 - 909) | 879  (821 - 937) | 26  (16 - 36) |
| Germany | Women | 1025  (962 - 1088) | 898  (839 - 957) | 782  (727 - 837) | 806  (750 - 862) | 24  (14 - 34) |
|  | Men | 586  (539 - 633) | 514  (470 - 558) | 447  (406 - 488) | 461  (419 - 503) | 14  (8 - 23) |
|  | Both sexes | 1611  (1532 - 1690) | 1411  (1337 - 1485) | 1229  (1160 - 1298) | 1267  (1197 - 1337) | 38  (26 - 50) |
| Greece | Women | 45  (32 - 58) | 39  (27 - 51) | 34  (23 - 45) | 35  (23 - 47) | 1  (0 - 6) |
|  | Men | 51  (37 - 65) | 45  (32 - 58) | 39  (27 - 51) | 40  (28 - 52) | 1  (0 - 6) |
|  | Both sexes | 96  (77 - 115) | 84  (66 - 102) | 73  (56 - 90) | 76  (59 - 93) | 2  (0 - 7) |
| Hungary | Women | 58  (43 - 73) | 51  (37 - 65) | 44  (31 - 57) | 46  (33 - 59) | 1  (0 - 6) |
|  | Men | 30  (19 - 41) | 26  (16 - 36) | 23  (14 - 32) | 24  (14 - 34) | 1  (0 - 6) |
|  | Both sexes | 88  (70 - 106) | 77  (60 - 94) | 67  (51 - 83) | 69  (53 - 85) | 2  (0 - 7) |
| Iceland | Women | 2  (0 - 7) | 2  (0 - 7) | 2  (0 - 7) | 2  (0 - 7) | 0  (0 - 4) |
|  | Men | 1  (0 - 6) | 1  (0 - 6) | 1  (0 - 6) | 1  (0 - 6) | 0  (0 - 4) |
|  | Both sexes | 4  (1 - 10) | 3  (1 - 9) | 3  (1 - 9) | 3  (1 - 9) | 0  (0 - 4) |
| Ireland | Women | 22  (13 - 31) | 19  (11 - 30) | 17  (10 - 27) | 17  (10 - 27) | 1  (0 - 6) |
|  | Men | 16  (9 - 26) | 14  (8 - 23) | 12  (6 - 21) | 12  (6 - 21) | 0  (0 - 4) |
|  | Both sexes | 38  (26 - 50) | 33  (22 - 44) | 29  (18 - 40) | 30  (19 - 41) | 1  (0 - 6) |
| Italy | Women | 601  (553 - 649) | 526  (481 - 571) | 458  (416 - 500) | 473  (430 - 516) | 14  (8 - 23) |
|  | Men | 418  (378 - 458) | 366  (329 - 403) | 319  (284 - 354) | 329  (293 - 365) | 10  (5 - 18) |
|  | Both sexes | 1019  (956 - 1082) | 892  (833 - 951) | 777  (722 - 832) | 801  (746 - 856) | 24  (14 - 34) |
| Latvia | Women | 10  (5 - 18) | 9  (4 - 17) | 8  (3 - 16) | 8  (3 - 16) | 0  (0 - 4) |
|  | Men | 6  (2 - 13) | 5  (2 - 12) | 5  (2 - 12) | 5  (2 - 12) | 0  (0 - 4) |
|  | Both sexes | 16  (9 - 26) | 14  (8 - 23) | 12  (6 - 21) | 13  (7 - 22) | 0  (0 - 4) |
| Lithuania | Women | 11  (5 - 20) | 9  (4 - 17) | 8  (3 - 16) | 8  (3 - 16) | 0  (0 - 4) |
|  | Men | 8  (3 - 16) | 7  (3 - 14) | 6  (2 - 13) | 6  (2 - 13) | 0  (0 - 4) |
|  | Both sexes | 18  (11 - 28) | 16  (9 - 26) | 14  (8 - 23) | 14  (8 - 23) | 0  (0 - 4) |
| Luxem-bourg | Women | 5  (2 - 12) | 5  (2 - 12) | 4  (1 - 10) | 4  (1 - 10) | 0  (0 - 4) |
|  | Men | 3  (1 - 9) | 3  (1 - 9) | 2  (0 - 7) | 2  (0 - 7) | 0  (0 - 4) |
|  | Both sexes | 8  (3 - 16) | 7  (3 - 14) | 6  (2 - 13) | 6  (2 - 13) | 0  (0 - 4) |
| Malta | Women | 2  (0 - 7) | 2  (0 - 7) | 1  (0 - 6) | 2  (0 - 7) | 0  (0 - 4) |
|  | Men | 1  (0 - 6) | 1  (0 - 6) | 1  (0 - 6) | 1  (0 - 6) | 0  (0 - 4) |
|  | Both sexes | 3  (1 - 9) | 2  (0 - 7) | 2  (0 - 7) | 2  (0 - 7) | 0  (0 - 4) |
| Norway | Women | 49  (35 - 63) | 43  (30 - 56) | 38  (26 - 50) | 39  (27 - 51) | 1  (0 - 6) |
|  | Men | 22  (13 - 31) | 19  (11 - 30) | 17  (10 - 27) | 17  (10 - 27) | 1  (0 - 6) |
|  | Both sexes | 72  (55 - 89) | 63  (47 - 79) | 55  (40 - 70) | 56  (41 - 71) | 2  (0 - 7) |
| Poland | Women | 232  (202 - 262) | 203  (175 - 231) | 177  (151 - 203) | 183  (156 - 210) | 5  (2 - 12) |
|  | Men | 113  (92 - 134) | 99  (79 - 119) | 86  (68 - 104) | 89  (71 - 107) | 3  (1 - 9) |
|  | Both sexes | 345  (309 - 381) | 302  (268 - 336) | 263  (231 - 295) | 271  (239 - 303) | 8  (3 - 16) |
| Portugal | Women | 84  (66 - 102) | 74  (57 - 91) | 64  (48 - 80) | 66  (50 - 82) | 2  (0 - 7) |
|  | Men | 38  (26 - 50) | 33  (22 - 44) | 29  (18 - 40) | 30  (19 - 41) | 1  (0 - 6) |
|  | Both sexes | 122  (100 - 144) | 107  (87 - 127) | 93  (74 - 112) | 96  (77 - 115) | 3  (1 - 9) |
| Romania | Women | 75  (58 - 92) | 66  (50 - 82) | 57  (42 - 72) | 59  (44 - 74) | 2  (0 - 7) |
|  | Men | 76  (59 - 93) | 66  (50 - 82) | 58  (43 - 73) | 59  (44 - 74) | 2  (0 - 7) |
|  | Both sexes | 150  (126 - 174) | 132  (109 - 155) | 115  (94 - 136) | 118  (97 - 139) | 4  (1 - 10) |
| Slovakia | Women | 23  (14 - 32) | 20  (11 - 29) | 18  (11 - 28) | 18  (11 - 28) | 1  (0 - 6) |
|  | Men | 15  (8 - 25) | 13  (7 - 22) | 11  (5 - 20) | 11  (5 - 20) | 0  (0 - 4) |
|  | Both sexes | 38  (26 - 50) | 33  (22 - 44) | 29  (18 - 40) | 30  (19 - 41) | 1  (0 - 6) |
| Slovenia | Women | 10  (5 - 18) | 9  (4 - 17) | 8  (3 - 16) | 8  (3 - 16) | 0  (0 - 4) |
|  | Men | 10  (5 - 18) | 9  (4 - 17) | 8  (3 - 16) | 8  (3 - 16) | 0  (0 - 4) |
|  | Both sexes | 20  (11 - 29) | 18  (11 - 28) | 16  (9 - 26) | 16  (9 - 26) | 0  (0 - 4) |
| Spain | Women | 173  (147 - 199) | 151  (127 - 175) | 132  (109 - 155) | 136  (113 - 159) | 4  (1 - 10) |
|  | Men | 217  (188 - 246) | 190  (163 - 217) | 166  (141 - 191) | 171  (145 - 197) | 5  (2 - 12) |
|  | Both sexes | 390  (351 - 429) | 342  (306 - 378) | 297  (263 - 331) | 307  (273 - 341) | 9  (4 - 17) |
| Sweden | Women | 98  (79 - 117) | 86  (68 - 104) | 75  (58 - 92) | 77  (60 - 94) | 2  (0 - 7) |
|  | Men | 44  (31 - 57) | 39  (27 - 51) | 34  (23 - 45) | 35  (23 - 47) | 1  (0 - 6) |
|  | Both sexes | 142  (119 - 165) | 124  (102 - 146) | 108  (88 - 128) | 111  (90 - 132) | 3  (1 - 9) |
| Switzer-land | Women | 142  (119 - 165) | 125  (103 - 147) | 108  (88 - 128) | 112  (91 - 133) | 3  (1 - 9) |
|  | Men | 55  (40 - 70) | 49 (35 - 63) | 42  (29 - 55) | 44  (31 - 57) | 1  (0 - 6) |
|  | Both sexes | 198  (170 - 226) | 173  (147 - 199) | 151  (127 - 175) | 155  (131 - 179) | 5  (2 - 12) |
| The Nether-lands | Women | 83  (65 - 101) | 73  (56 - 90) | 63  (47 - 79) | 65  (49 - 81) | 2  (0 - 7) |
|  | Men | 68  (52 - 84) | 60  (45 - 75) | 52  (38 - 66) | 53  (39 - 67) | 2  (0 - 7) |
|  | Both sexes | 151  (127 - 175) | 132  (109 - 155) | 115  (94 - 136) | 119  (98 - 140) | 4  (1 - 10) |
| United Kingdom | Women | 597  (549 - 645) | 523  (478 - 568) | 455  (413 - 497) | 469  (427 - 511) | 14  (8 - 23) |
|  | Men | 400  (361 - 439) | 350  (313 - 387) | 305  (271 - 339) | 314  (279 - 349) | 9  (4 - 17) |
|  | Both sexes | 996  (934 - 1058) | 873  (815 - 931) | 760  (706 - 814) | 784  (729 - 839) | 24  (14 - 34) |

^10^ HPV prevalence: 87.6% (81.6–92.1), ^11^ HPV 16/18 attributable fraction among HPV+ cases: 87.1% (80.7–92.1), ^12^ HPV 31/33/45/52/558 attributable fraction among HPV+ cases: 89.8% (83.8–94.2) (ref: Alemany et al [19])

HPV: human papillomavirus; CI: confidence interval.
