# Supplementary material for: Estimation of the epidemiological burden of HPV-related anogenital cancers, precancerous lesions, and genital warts in women and men in Europe: Potential additional benefit of a nine-valent second generation HPV vaccine compared to first generation HPV vaccines
Source: Papillomavirus Res. 2015 Jun 16;1:90–100. doi: 10.1016/j.pvr.2015.06.003 (PMC5886848; doi:10.1016/j.pvr.2015.06.003)
Supplement: Supplementary file 2 — Supplementary material [file mmc2.docx]

**Table 4 - Estimated annual number of new precancerous lesions in women and men in the European countries**

1. **CIN2+**

| **Country** | **N of new cases irrespective of HPV status (range)** | **N of new cases attributable to HPV (range)^1^** | **N of cases related to quadrivalent vaccine types (range)^2^** | **N of cases related to nine-valent vaccine types (range)^3^** | **Additional N of cases related to five new HPV types (range)** |
| --- | --- | --- | --- | --- | --- |
| Austria | 4422 - 8422 | 4422 - 8422 | 2012 - 3832 | 3639 - 6932 | 1627 - 3099 |
| Belgium | 5632 - 10802 | 5632 - 10802 | 2562 - 4915 | 4635 - 8890 | 2072 - 3975 |
| Bulgaria | 3762 - 7262 | 3762 - 7262 | 1712 - 3304 | 3096 - 5977 | 1384 - 2673 |
| Croatia | 2151 - 4135 | 2151 - 4135 | 979 - 1882 | 1771 - 3403 | 792 - 1522 |
| Cyprus | 532 - 1032 | 532 - 1032 | 242 - 469 | 438 - 849 | 196 - 380 |
| Czech Republic | 5695 - 10931 | 5695 - 10931 | 2591 - 4973 | 4687 - 8996 | 2096 - 4023 |
| Denmark | 2738 - 5251 | 2738 - 5251 | 1246 - 2389 | 2254 - 4322 | 1008 - 1932 |
| Estonia | 700 - 1357 | 700 - 1357 | 318 - 617 | 576 - 1116 | 257 - 499 |
| Finland | 2598 - 5043 | 2598 - 5043 | 1182 - 2295 | 2138 - 4151 | 956 - 1856 |
| France | 32263 - 61861 | 32263 - 61861 | 14679 - 28147 | 26552 - 50912 | 11873 - 22765 |
| Germany | 40242 - 76655 | 40242 - 76655 | 18310 - 34878 | 33119 - 63087 | 14809 - 28209 |
| Greece | 5680 - 10764 | 5680 - 10764 | 2584 - 4897 | 4674 - 8858 | 2090 - 3961 |
| Hungary | 5260 - 10072 | 5260 - 10072 | 2393 - 4583 | 4329 - 8289 | 1936 - 3707 |
| Iceland | 169 - 327 | 169 - 327 | 77 - 149 | 139 - 269 | 62 - 120 |
| Ireland | 2539 - 4826 | 2539 - 4826 | 1155 - 2196 | 2089 - 3972 | 934 - 1776 |
| Italy | 29603 - 55625 | 29603 - 55625 | 13469 - 25309 | 24363 - 45779 | 10894 - 20470 |
| Latvia | 1089 - 2110 | 1089 - 2110 | 495 - 960 | 896 - 1737 | 401 - 776 |
| Lithuania | 1553 - 2993 | 1553 - 2993 | 707 - 1362 | 1278 - 2463 | 572 - 1102 |
| Luxembourg | 293 - 555 | 293 - 555 | 133 - 253 | 241 - 457 | 108 - 204 |
| Malta | 220 - 430 | 220 - 430 | 100 - 196 | 181 - 354 | 81 - 158 |
| Norway | 2567 - 4933 | 2567 - 4933 | 1168 - 2244 | 2113 - 4060 | 945 - 1815 |
| Poland | 21847 - 42462 | 21847 - 42462 | 9940 - 19320 | 17980 - 34946 | 8040 - 15626 |
| Portugal | 5457 - 10315 | 5457 - 10315 | 2483 - 4693 | 4491 - 8490 | 2008 - 3796 |
| Romania | 10578 - 20246 | 10578 - 20246 | 4813 - 9212 | 8706 - 16662 | 3893 - 7450 |
| Slovakia | 3123 - 6031 | 3123 - 6031 | 1421 - 2744 | 2570 - 4964 | 1149 - 2219 |
| Slovenia | 1061 - 2022 | 1061 - 2022 | 483 - 920 | 873 - 1664 | 390 - 744 |
| Spain | 25035 - 46996 | 25035 - 46996 | 11391 - 21383 | 20604 - 38678 | 9213 - 17295 |
| Sweden | 4753 - 9199 | 4753 - 9199 | 2163 - 4186 | 3912 - 7571 | 1749 - 3385 |
| Switzerland | 4231 - 8052 | 4231 - 8052 | 1925 - 3664 | 3482 - 6627 | 1557 - 2963 |
| The Netherlands | 8326 - 15934 | 8326 - 15934 | 3788 - 7250 | 6852 - 13113 | 3064 - 5864 |
| United Kingdom | 33230 - 63965 | 33230 - 63965 | 15120 - 29104 | 27349 - 52643 | 12229 - 23539 |

^1^ HPV prevalence: 100%, ^2^ HPV6/11/16/18 attributable fraction among HPV+ cases: 45.5%, ^3^ HPV 6/11/16/18/31/33/45/52/58 attributable fraction among HPV+ cases: 82.3% ref: Joura et al. [17] with additional information from authors

1. **VIN2/3**

| **Country** | **N of new cases irrespective of HPV status (range)** | **N of new cases attributable to HPV (range)^4^** | **N of cases related to quadrivalent vaccine types (range)^5^** | **N of cases related to nine-valent vaccine types (range)^6^** | **Additional N of cases related to five new HPV types (range)** |
| --- | --- | --- | --- | --- | --- |
| Austria | 229 - 454 | 199 - 394 | 164 - 324 | 188 - 372 | 24 - 48 |
| Belgium | 288 - 574 | 250 - 499 | 206 - 410 | 236 - 471 | 31 - 61 |
| Bulgaria | 202 - 406 | 175 - 353 | 144 - 290 | 165 - 333 | 21 - 43 |
| Croatia | 116 - 229 | 101 - 199 | 83 - 164 | 95 - 188 | 12 - 24 |
| Cyprus | 22 - 45 | 19 - 39 | 15 - 32 | 18 - 37 | 2 - 5 |
| Czech Republic | 280 - 571 | 244 - 496 | 200 - 408 | 230 - 468 | 30 - 61 |
| Denmark | 143 - 288 | 124 - 251 | 102 - 206 | 117 - 237 | 15 - 31 |
| Estonia | 37 - 73 | 32 - 63 | 26 - 52 | 30 - 60 | 4 - 8 |
| Finland | 141 - 285 | 123 - 248 | 101 - 203 | 116 - 234 | 15 - 30 |
| France | 1691 - 3370 | 1470 - 2928 | 1208 - 2407 | 1387 - 2764 | 179 - 357 |
| Germany | 2274 - 4431 | 1976 - 3850 | 1624 - 3165 | 1865 - 3635 | 241 - 470 |
| Greece | 299 - 590 | 260 - 512 | 214 - 421 | 246 - 484 | 32 - 63 |
| Hungary | 275 - 550 | 239 - 478 | 196 - 393 | 225 - 451 | 29 - 58 |
| Iceland | 7 - 15 | 6 - 13 | 5 - 11 | 6 - 12 | 1 - 2 |
| Ireland | 108 - 220 | 94 - 191 | 77 - 157 | 89 - 181 | 11 - 23 |
| Italy | 1681 - 3270 | 1461 - 2841 | 1201 - 2336 | 1379 - 2682 | 178 - 347 |
| Latvia | 58 - 116 | 51 - 101 | 42 - 83 | 48 - 95 | 6 - 12 |
| Lithuania | 85 - 167 | 74 - 145 | 60 - 119 | 69 - 137 | 9 - 18 |
| Luxembourg | 14 - 27 | 12 - 23 | 10 - 19 | 11 - 22 | 1 - 3 |
| Malta | 11 - 22 | 9 - 19 | 8 - 16 | 9 - 18 | 1 - 2 |
| Norway | 123 - 250 | 107 - 217 | 88 - 178 | 101 - 205 | 13 - 26 |
| Poland | 1014 - 2066 | 882 - 1795 | 725 - 1476 | 832 - 1695 | 108 - 219 |
| Portugal | 293 - 577 | 255 - 502 | 209 - 412 | 241 - 474 | 31 - 61 |
| Romania | 532 - 1066 | 462 - 926 | 380 - 762 | 436 - 875 | 56 - 113 |
| Slovakia | 141 - 289 | 123 - 251 | 101 - 207 | 116 - 237 | 15 - 31 |
| Slovenia | 55 - 109 | 48 - 95 | 40 - 78 | 45 - 90 | 6 - 12 |
| Spain | 1255 - 2471 | 1090 - 2147 | 896 - 1765 | 1029 - 2027 | 133 - 262 |
| Sweden | 241 - 487 | 209 - 423 | 172 - 348 | 197 - 400 | 26 - 52 |
| Switzerland | 214 - 427 | 186 - 371 | 153 - 305 | 176 - 350 | 23 - 45 |
| The Netherlands | 436 - 874 | 378 - 760 | 311 - 624 | 357 - 717 | 46 - 93 |
| United Kingdom | 1620 - 3273 | 1408 - 2844 | 1157 - 2338 | 1329 - 2685 | 172 - 347 |

^4^ HPV prevalence: 86.9% 82.6– 90.4, ^5^ HPV6/11/16/18 attributable fraction among HPV+ cases: 82.2% 77.2–86.6, ^6^ HPV 6/11/16/18/31/33/45/52/58 attributable fraction among HPV+ cases: 94.4% 91.0–96.9 ref: de Sanjosé et al. [18]

1. **VaIN 2/3**

| **Country** | **N of new cases irrespective of HPV status (range)** | **N of new cases attributable to HPV (range)^7^** | **N of cases related to quadrivalent vaccine types (range)^8^** | **N of cases related to nine-valent vaccine types (range)^9^** | **Additional N of cases related to five new HPV types (range)** |
| --- | --- | --- | --- | --- | --- |
| Austria | 42 - 78 | 40 - 75 | 26 - 48 | 31 - 58 | 5 - 10 |
| Belgium | 53 - 98 | 51 - 94 | 32 - 60 | 39 - 73 | 7 - 13 |
| Bulgaria | 38 - 69 | 37 - 66 | 23 - 42 | 28 - 51 | 5 - 9 |
| Croatia | 22 - 39 | 21 - 38 | 13 - 24 | 16 - 29 | 3 - 5 |
| Cyprus | 4 - 7 | 3 - 7 | 2 - 5 | 3 - 6 | 0 - 1 |
| Czech Republic | 51 - 95 | 49 - 91 | 31 - 59 | 38 - 71 | 7 - 12 |
| Denmark | 26 - 49 | 25 - 47 | 16 - 30 | 20 - 36 | 3 - 6 |
| Estonia | 7 - 13 | 7 - 12 | 4 - 8 | 5 - 9 | 1 - 2 |
| Finland | 27 - 48 | 26 - 46 | 17 - 30 | 20 - 36 | 3 - 6 |
| France | 314 - 576 | 301 - 552 | 193 - 354 | 233 - 428 | 41 - 74 |
| Germany | 432 - 772 | 414 - 740 | 265 - 474 | 321 - 574 | 56 - 100 |
| Greece | 55 - 102 | 53 - 98 | 34 - 63 | 41 - 76 | 7 - 13 |
| Hungary | 51 - 93 | 49 - 89 | 31 - 57 | 38 - 69 | 7 - 12 |
| Iceland | 1 - 3 | 1 - 2 | 1 - 2 | 1 - 2 | 0 - 0 |
| Ireland | 17 - 36 | 17 - 35 | 11 - 22 | 13 - 27 | 2 - 5 |
| Italy | 317 - 572 | 304 - 548 | 195 - 351 | 236 - 425 | 41 - 74 |
| Latvia | 11 - 20 | 11 - 19 | 7 - 12 | 8 - 15 | 1 - 3 |
| Lithuania | 16 - 29 | 15 - 28 | 10 - 18 | 12 - 21 | 2 - 4 |
| Luxembourg | 2 - 5 | 2 - 4 | 1 - 3 | 2 - 3 | 0 - 1 |
| Malta | 2 - 4 | 2 - 4 | 1 - 2 | 1 - 3 | 0 - 0 |
| Norway | 22 - 42 | 21 - 40 | 13 - 26 | 16 - 31 | 3 - 5 |
| Poland | 179 - 343 | 172 - 328 | 110 - 211 | 133 - 255 | 23 - 44 |
| Portugal | 54 - 99 | 52 - 95 | 33 - 61 | 40 - 74 | 7 - 13 |
| Romania | 96 - 180 | 92 - 173 | 59 - 111 | 72 - 134 | 12 - 23 |
| Slovakia | 24 - 48 | 23 - 46 | 15 - 29 | 18 - 35 | 3 - 6 |
| Slovenia | 10 - 19 | 10 - 18 | 6 - 11 | 8 - 14 | 1 - 2 |
| Spain | 222 - 425 | 213 - 407 | 136 - 261 | 165 - 316 | 29 - 55 |
| Sweden | 45 - 83 | 43 - 79 | 28 - 51 | 33 - 62 | 6 - 11 |
| Switzerland | 38 - 73 | 37 - 70 | 24 - 45 | 29 - 54 | 5 - 9 |
| The Netherlands | 79 - 147 | 76 - 141 | 49 - 90 | 59 - 109 | 10 - 19 |
| United Kingdom | 291 - 553 | 279 - 530 | 179 - 340 | 217 - 411 | 38 - 72 |

^7^ HPV prevalence: 95.8% 91.8– 98.2, ^8^ HPV6/11/16/18 attributable fraction among HPV+ cases: 64.1% 56.6–71.2, ^3^ HPV 6/11/16/18/31/33/45/52/58 attributable fraction among HPV+ cases: 77.6% 70.6–83.3 ref: Alemany et al. [25]

**AIN 2/3**

| **Country** | **Sexes** | **N of new cases irrespective of HPV status** | **N of new cases attributable to HPV** | **N of cases related to quadrivalent vaccine types** | **N of cases related to nine-valent vaccine types** | **Additional N of cases related to five new HPV types** |
| --- | --- | --- | --- | --- | --- | --- |
| Austria | Women | 25 | 24 | 18 | 20 | 1 |
|  | Men | 18 | 17 | 13 | 14 | 1 |
|  | Both sexes | 43 | 41 | 31 | 33 | 2 |
| Belgium | Women | 33 | 31 | 24 | 26 | 2 |
|  | Men | 24 | 22 | 17 | 18 | 1 |
|  | Both sexes | 57 | 54 | 41 | 44 | 3 |
| Bulgaria | Women | 22 | 21 | 16 | 17 | 1 |
|  | Men | 15 | 15 | 11 | 12 | 1 |
|  | Both sexes | 37 | 35 | 27 | 29 | 2 |
| Croatia | Women | 13 | 12 | 9 | 10 | 1 |
|  | Men | 9 | 8 | 6 | 7 | 1 |
|  | Both sexes | 22 | 21 | 16 | 17 | 1 |
| Cyprus | Women | 3 | 2 | 2 | 2 | 0 |
|  | Men | 2 | 2 | 1 | 1 | 0 |
|  | Both sexes | 4 | 4 | 3 | 3 | 0 |
| Czech Republic | Women | 31 | 30 | 22 | 24 | 2 |
|  | Men | 22 | 21 | 16 | 17 | 1 |
|  | Both sexes | 53 | 51 | 38 | 41 | 3 |
| Denmark | Women | 16 | 16 | 12 | 13 | 1 |
|  | Men | 12 | 11 | 9 | 9 | 1 |
|  | Both sexes | 28 | 27 | 20 | 22 | 2 |
| Estonia | Women | 4 | 4 | 3 | 3 | 0 |
|  | Men | 3 | 3 | 2 | 2 | 0 |
|  | Both sexes | 7 | 6 | 5 | 5 | 0 |
| Finland | Women | 16 | 15 | 12 | 12 | 1 |
|  | Men | 11 | 11 | 8 | 9 | 1 |
|  | Both sexes | 27 | 26 | 20 | 21 | 2 |
| France | Women | 196 | 187 | 141 | 152 | 11 |
|  | Men | 137 | 130 | 98 | 106 | 8 |
|  | Both sexes | 333 | 317 | 239 | 258 | 19 |
| Germany | Women | 242 | 230 | 174 | 188 | 14 |
|  | Men | 173 | 165 | 125 | 135 | 10 |
|  | Both sexes | 415 | 396 | 298 | 322 | 24 |
| Greece | Women | 33 | 31 | 24 | 25 | 2 |
|  | Men | 23 | 22 | 17 | 18 | 1 |
|  | Both sexes | 56 | 53 | 40 | 44 | 3 |
| Hungary | Women | 30 | 29 | 22 | 23 | 2 |
|  | Men | 20 | 19 | 15 | 16 | 1 |
|  | Both sexes | 50 | 48 | 36 | 39 | 3 |
| Iceland | Women | 1 | 1 | 1 | 1 | 0 |
|  | Men | 1 | 1 | 1 | 1 | 0 |
|  | Both sexes | 2 | 2 | 1 | 1 | 0 |
| Ireland | Women | 13 | 13 | 10 | 10 | 1 |
|  | Men | 10 | 9 | 7 | 8 | 1 |
|  | Both sexes | 23 | 22 | 17 | 18 | 1 |
| Italy | Women | 179 | 170 | 128 | 139 | 10 |
|  | Men | 124 | 118 | 89 | 96 | 7 |
|  | Both sexes | 303 | 289 | 218 | 235 | 18 |
| Latvia | Women | 6 | 6 | 5 | 5 | 0 |
|  | Men | 4 | 4 | 3 | 3 | 0 |
|  | Both sexes | 10 | 10 | 7 | 8 | 1 |
| Lithuania | Women | 9 | 9 | 7 | 7 | 1 |
|  | Men | 6 | 6 | 4 | 5 | 0 |
|  | Both sexes | 15 | 14 | 11 | 12 | 1 |
| Luxembourg | Women | 2 | 1 | 1 | 1 | 0 |
|  | Men | 1 | 1 | 1 | 1 | 0 |
|  | Both sexes | 3 | 3 | 2 | 2 | 0 |
| Malta | Women | 1 | 1 | 1 | 1 | 0 |
|  | Men | 1 | 1 | 1 | 1 | 0 |
|  | Both sexes | 2 | 2 | 2 | 2 | 0 |
| Norway | Women | 15 | 14 | 10 | 11 | 1 |
|  | Men | 11 | 10 | 8 | 8 | 1 |
|  | Both sexes | 25 | 24 | 18 | 20 | 1 |
| Poland | Women | 115 | 110 | 83 | 90 | 7 |
|  | Men | 80 | 76 | 58 | 62 | 5 |
|  | Both sexes | 196 | 186 | 140 | 152 | 11 |
| Portugal | Women | 32 | 30 | 23 | 25 | 2 |
|  | Men | 21 | 20 | 15 | 17 | 1 |
|  | Both sexes | 53 | 51 | 38 | 41 | 3 |
| Romania | Women | 60 | 57 | 43 | 46 | 3 |
|  | Men | 42 | 40 | 30 | 33 | 2 |
|  | Both sexes | 101 | 97 | 73 | 79 | 6 |
| Slovakia | Women | 16 | 15 | 12 | 13 | 1 |
|  | Men | 11 | 11 | 8 | 9 | 1 |
|  | Both sexes | 27 | 26 | 20 | 21 | 2 |
| Slovenia | Women | 6 | 6 | 4 | 5 | 0 |
|  | Men | 4 | 4 | 3 | 3 | 0 |
|  | Both sexes | 10 | 10 | 7 | 8 | 1 |
| Spain | Women | 138 | 131 | 99 | 107 | 8 |
|  | Men | 99 | 94 | 71 | 77 | 6 |
|  | Both sexes | 237 | 225 | 170 | 184 | 14 |
| Sweden | Women | 28 | 26 | 20 | 22 | 2 |
|  | Men | 20 | 20 | 15 | 16 | 1 |
|  | Both sexes | 48 | 46 | 35 | 38 | 3 |
| Switzerland | Women | 24 | 23 | 17 | 18 | 1 |
|  | Men | 17 | 16 | 12 | 13 | 1 |
|  | Both sexes | 41 | 39 | 29 | 32 | 2 |
| The Netherlands | Women | 49 | 47 | 35 | 38 | 3 |
|  | Men | 36 | 34 | 26 | 28 | 2 |
|  | Both sexes | 85 | 81 | 61 | 66 | 5 |
| United Kingdom | Women | 188 | 179 | 135 | 146 | 11 |
|  | Men | 135 | 129 | 97 | 105 | 8 |
|  | Both sexes | 323 | 308 | 232 | 251 | 19 |

^10^ HPV prevalence: 87.6% 81.6–92.1, ^11^ HPV6/11/16/18 attributable fraction among HPV+ cases: 87.1% 80.7–92.1, ^12^ HPV 6/11/16/18/31/33/45/52/58 attributable fraction among HPV+ cases: 89.8% 83.8–94.2 ref: Alemany et al. [19]

HPV: human papillomavirus; CIN: cervical intraepithelial neoplasia; CIN2+ includes CIN2, CIN3 and AIS, VIN: vulvar intraepithelial neoplasia; VaIN: vaginal intraepithelial neoplasia; AIN: anal intraepithelial neoplasia; N: number
