# Supplementary material for: Estimation of the epidemiological burden of HPV-related anogenital cancers, precancerous lesions, and genital warts in women and men in Europe: Potential additional benefit of a nine-valent second generation HPV vaccine compared to first generation HPV vaccines
Source: Papillomavirus Res. 2015 Jun 16;1:90–100. doi: 10.1016/j.pvr.2015.06.003 (PMC5886848; doi:10.1016/j.pvr.2015.06.003)
Supplement: Supplementary file 3 — Supplementary material [file mmc3.docx]

**Table 5 - Estimated annual number of new genital wart cases in women and men in the European countries**

| **Country** | **Sex** | **N of new annual cases (range)** | **N of new annual cases related to HPV6/11 (range)** |
| --- | --- | --- | --- |
| Austria | Women | 6146 - 8271 | 5531 - 7444 |
|  | Men | 6089 - 6915 | 5480 - 6224 |
|  | Both sexes | 12235 - 15187 | 11012 - 13668 |
| Belgium | Women | 8057 - 10843 | 7252 - 9759 |
|  | Men | 8103 - 9202 | 7292 - 8282 |
|  | Both sexes | 16160 - 20046 | 14544 - 18041 |
| Bulgaria | Women | 5310 - 7146 | 4779 - 6432 |
|  | Men | 5235 - 5945 | 4711 - 5351 |
|  | Both sexes | 10545 - 13091 | 9490 - 11782 |
| Croatia | Women | 3132 - 4215 | 2819 - 3794 |
|  | Men | 3037 - 3449 | 2733 - 3104 |
|  | Both sexes | 6169 - 7664 | 5552 - 6897 |
| Cyprus | Women | 632 - 850 | 569 - 765 |
|  | Men | 622 - 706 | 560 - 635 |
|  | Both sexes | 1253 - 1556 | 1128 - 1401 |
| Czech Republic | Women | 7600 - 10227 | 6840 - 9205 |
|  | Men | 7626 - 8661 | 6863 - 7795 |
|  | Both sexes | 15225 - 18888 | 13703 - 16999 |
| Denmark | Women | 4010 - 5396 | 3609 - 4857 |
|  | Men | 4103 - 4660 | 3693 - 4194 |
|  | Both sexes | 8113 - 10056 | 7302 - 9051 |
| Estonia | Women | 1000 - 1345 | 900 - 1211 |
|  | Men | 910 - 1033 | 819 - 930 |
|  | Both sexes | 1910 - 2379 | 1719 - 2141 |
| Finland | Women | 3919 - 5274 | 3527 - 4747 |
|  | Men | 3938 - 4472 | 3544 - 4025 |
|  | Both sexes | 7857 - 9746 | 7071 - 8772 |
| France | Women | 48016 - 64619 | 43215 - 58157 |
|  | Men | 46904 - 53269 | 42213 - 47942 |
|  | Both sexes | 94920 - 117888 | 85428 - 106099 |
| Germany | Women | 59177 - 79638 | 53259 - 71675 |
|  | Men | 59576 - 67662 | 53619 - 60896 |
|  | Both sexes | 118753 - 147300 | 106878 - 132570 |
| Greece | Women | 8022 - 10796 | 7220 - 9716 |
|  | Men | 7993 - 9078 | 7194 - 8170 |
|  | Both sexes | 16015 - 19874 | 14414 - 17886 |
| Hungary | Women | 7374 - 9924 | 6636 - 8931 |
|  | Men | 6964 - 7909 | 6267 - 7118 |
|  | Both sexes | 14337 - 17832 | 12904 - 16049 |
| Iceland | Women | 228 - 307 | 205 - 276 |
|  | Men | 238 - 271 | 215 - 244 |
|  | Both sexes | 466 - 577 | 420 - 520 |
| Ireland | Women | 3292 - 4430 | 2963 - 3987 |
|  | Men | 3356 - 3811 | 3020 - 3430 |
|  | Both sexes | 6648 - 8242 | 5983 - 7418 |
| Italy | Women | 43730 - 58850 | 39357 - 52965 |
|  | Men | 42658 - 48448 | 38393 - 43603 |
|  | Both sexes | 86388 - 107298 | 77749 - 96568 |
| Latvia | Women | 1558 - 2097 | 1402 - 1887 |
|  | Men | 1368 - 1554 | 1231 - 1398 |
|  | Both sexes | 2926 - 3651 | 2634 - 3286 |
| Lithuania | Women | 2276 - 3063 | 2049 - 2757 |
|  | Men | 2021 - 2296 | 1819 - 2066 |
|  | Both sexes | 4298 - 5359 | 3868 - 4823 |
| Luxembourg | Women | 381 - 513 | 343 - 462 |
|  | Men | 396 - 450 | 357 - 405 |
|  | Both sexes | 778 - 963 | 700 - 867 |
| Malta | Women | 300 - 404 | 270 - 364 |
|  | Men | 310 - 352 | 279 - 317 |
|  | Both sexes | 610 - 756 | 549 - 681 |
| Norway | Women | 3572 - 4807 | 3215 - 4326 |
|  | Men | 3745 - 4253 | 3370 - 3827 |
|  | Both sexes | 7316 - 9060 | 6585 - 8154 |
| Poland | Women | 28235 - 37998 | 25412 - 34198 |
|  | Men | 27538 - 31275 | 24784 - 28147 |
|  | Both sexes | 55773 - 69273 | 50196 - 62346 |
| Portugal | Women | 7798 - 10494 | 7018 - 9445 |
|  | Men | 7377 - 8378 | 6639 - 7540 |
|  | Both sexes | 15175 - 18872 | 13657 - 16985 |
| Romania | Women | 14567 - 19604 | 13110 - 17644 |
|  | Men | 14414 - 16370 | 12972 - 14733 |
|  | Both sexes | 28981 - 35974 | 26083 - 32377 |
| Slovakia | Women | 3940 - 5303 | 3546 - 4772 |
|  | Men | 3892 - 4421 | 3503 - 3978 |
|  | Both sexes | 7833 - 9723 | 7049 - 8751 |
| Slovenia | Women | 1476 - 1987 | 1329 - 1788 |
|  | Men | 1505 - 1709 | 1354 - 1538 |
|  | Both sexes | 2981 - 3696 | 2683 - 3326 |
| Spain | Women | 33668 - 45310 | 30302 - 40779 |
|  | Men | 33988 - 38601 | 30589 - 34741 |
|  | Both sexes | 67656 - 83911 | 60891 - 75520 |
| Sweden | Women | 6802 - 9154 | 6122 - 8238 |
|  | Men | 7037 - 7992 | 6334 - 7193 |
|  | Both sexes | 13839 - 17146 | 12455 - 15431 |
| Switzerland | Women | 5780 - 7779 | 5202 - 7001 |
|  | Men | 5860 - 6655 | 5274 - 5990 |
|  | Both sexes | 11640 - 14434 | 10476 - 12991 |
| The Netherlands | Women | 12031 - 16190 | 10828 - 14571 |
|  | Men | 12267 - 13931 | 11040 - 12538 |
|  | Both sexes | 24297 - 30122 | 21867 - 27110 |
| United Kingdom | Women | 46111 - 62055 | 41500 - 55850 |
|  | Men | 46400 - 52697 | 41760 - 47427 |
|  | Both sexes | 92511 - 114752 | 83260 - 103277 |

HPV: human papillomavirus; N: number.
